# Transitioning a Large Scale HIV/AIDS Prevention Program to Local Stakeholders: Findings from the Avahan Transition Evaluation

**DOI:** 10.1371/journal.pone.0136177

**Published:** 2015-09-01

**Authors:** Sara Bennett, Suneeta Singh, Daniela Rodriguez, Sachiko Ozawa, Kriti Singh, Vibha Chhabra, Neeraj Dhingra

**Affiliations:** 1 Johns Hopkins Bloomberg School of Public Health, International Health Department, 615 North Wolfe St, Baltimore, MD, United States of America; 2 Amaltas Consulting Pvt. Ltd., C-20 Hauz Khas, New Delhi, 110016, India; 3 Department of Aids Control, Ministry of Health & Family Welfare, Government of India, 6^th^ Floor, Chandralok Building, 36 Janpath, New Delhi, 110001, India; British Columbia Centre for Excellence in HIV/AIDS, CANADA

## Abstract

**Background:**

Between 2009–2013 the Bill and Melinda Gates Foundation transitioned its HIV/AIDS prevention initiative in India from being a stand-alone program outside of government, to being fully government funded and implemented. We present an independent prospective evaluation of the transition.

**Methods:**

The evaluation drew upon (1) a structured survey of transition readiness in a sample of 80 targeted HIV prevention programs prior to transition; (2) a structured survey assessing institutionalization of program features in a sample of 70 targeted intervention (TI) programs, one year post-transition; and (3) case studies of 15 TI programs.

**Findings:**

Transition was conducted in 3 rounds. While the 2009 transition round was problematic, subsequent rounds were implemented more smoothly. In the 2011 and 2012 transition rounds, Avahan programs were well prepared for transition with the large majority of TI program staff trained for transition, high alignment with government clinical, financial and managerial norms, and strong government commitment to the program. One year post transition there were significant program changes, but these were largely perceived positively. Notable negative changes were: limited flexibility in program management, delays in funding, commodity stock outs, and community member perceptions of a narrowing in program focus. Service coverage outcomes were sustained at least six months post-transition.

**Interpretation:**

The study suggests that significant investments in transition preparation contributed to a smooth transition and sustained service coverage. Notwithstanding, there were substantive program changes post-transition. Five key lessons for transition design and implementation are identified.

## Introduction

Recent global policy statements have reinforced the centrality of country ownership and leadership of the HIV/AIDS response[1,2]. Program transition, that is the formal handing over of a program to one or more local partners so as to sustain key elements of the program over time, might be seen as the ultimate articulation of country leadership. While academic literature on program transition and sustainability in high income countries has attracted considerable interest[3–6] there has been very limited empirical enquiry into these issues in low and middle income countries[7,8], and those studies which are available are largely retrospective case studies. We present a prospective assessment of the transition of the Avahan India HIV/AIDS initiative.

Between 2009–2013 the Bill and Melinda Gates Foundation (BMGF) transitioned its major HIV/AIDS prevention initiative in India from being a stand-alone program outside of government, albeit coordinated with government, to being a fully government funded, managed and implemented program[9]. The experience of managing program transition was documented by BMGF staff and a number of lessons identified, namely: the importance of an in-country team to develop and nurture relationships with local partners; the importance of monitoring and support to programs post-transition; dedicated funding to support transition activities; and an extended time frame to allow for learning between tranches[9]. This paper reports the findings of an independent evaluation of the Avahan transition process.

The Avahan India HIV/AIDS Initiative funded by BMGF, was started in 2003 and was rolled out in the states of Karnataka, Andhra Pradesh, Tamil Nadu, Maharashtra, Manipur and Nagaland in India. During the first phase (2003–08) the goal of the program was to build and operate an HIV prevention program at scale for high risk groups (HRGs). Avahan was implemented through a “parallel” system of non-governmental organizations (NGOs) and community based organizations (CBOs) that facilitated achievement of scale very rapidly[10]. In total, Avahan provided funding and support to approximately 200 targeted HIV prevention interventions (TIs) across more than 600 towns in 82 districts in six states, covering approximately 200,000 female sex workers (FSW), 80,000 men who have sex with men (MSM) and transgenders, and 20,000 intravenous drug users[10]. It offered a standardized package of services including outreach and behavior change communication, commodity distribution (condoms), linkages to care and treatment, clinical services for sexually transmitted infections (STI), community mobilization and creation of an enabling environment for HRGs through advocacy and crisis response[11].

**Fig 1:**
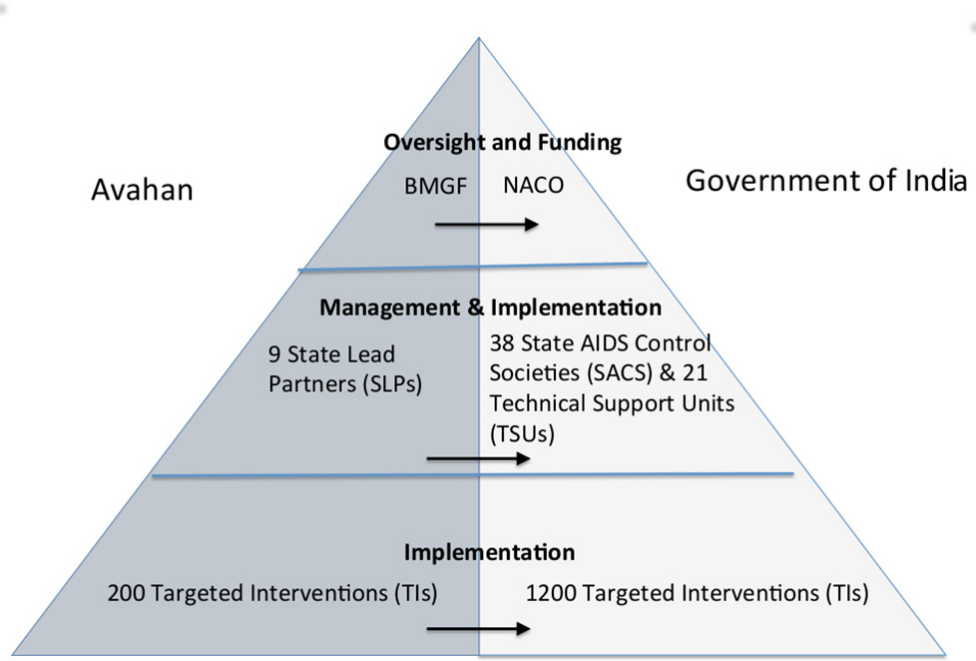
Organizational arrangements for the transition of the Avahan initiative to government.

During the second phase (2009–2013) BMGF transferred the programs to the Government of India (Fig 1). Avahan handed high-level responsibility for management and funding of the program over to the National AIDS Control Organization (NACO). State Lead Partners (SLPs), typically large international or national NGOs, handed over their management and implementation responsibilities to State AIDS Control Societies (SACS) and their associated Technical Support Units (TSU). Implementation of program activities continued to be conducted by much the same pool of NGOs and community based organizations (CBOs), now under contract to government rather than Avahan. The transition process occurred in three rounds, with 10% of TI programs transitioning in 2009, 20% in 2011 and the remaining 70% in 2012. While Avahan made a significant contribution to the scale-up effort, by 2009 government funded and managed TI programs numbered approximately 1200, and combined, government and donors covered 53% of FSWs, 78% of MSM and 74% of IDUs [12].

This evaluation aimed to generate practical lessons concerning the transition of Avahan to local ownership, so as to guide implementation strategies throughout the transition process, and inform similar processes elsewhere. Specifically we addressed the following questions:-

- What was the Avahan transition strategy and how was it implemented?
- How well prepared were the TI programs for transition?
- How well institutionalized is the Avahan program post-transition?
- Were program effects sustained after transition?
- What are the elements necessary for effective transition of HIV/AIDS prevention programs?

This paper synthesizes findings from across the entire evaluation.

## Methods

**Fig 2:**
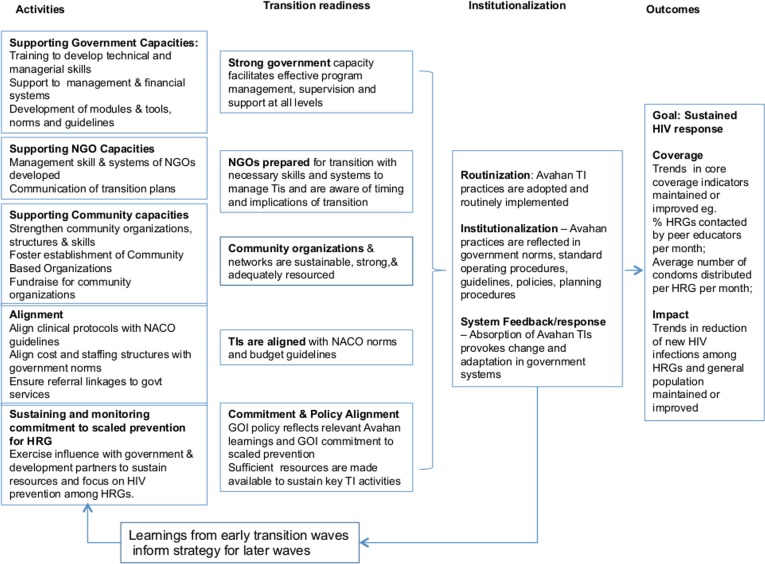
Avahan transition evaluation logic model.

The logic model for the transition evaluation (Fig 2) posited that Avahan undertook a range of activities to prepare the program for transition, notably (i) developing capacity of partners (government, communities and NGOs) (ii) aligning Avahan interventions with government norms and guidelines, and (iii) monitoring commitment of government and other actors to the program. These preparatory activities led to a state of “transition readiness” that facilitated the transition process, which in turn supported the institutionalization of key Avahan practices and a sustained HIV/AIDS response[13].

During 2009–2013 we conducted a mixed methods evaluation with multiple rounds of data collection (corresponding to the different transition rounds) that combined surveys of transitioning TIs both just before they transitioned, and approximately one year after transition, with qualitative case studies of purposively selected TI programs. This paper focuses on the TIs that transitioned in the Southern states of Karnataka, Andhra Pradesh, Tamil Nadu and Maharashtra.

A transition readiness survey was conducted among all 27 TI programs that transitioned in 2011 and a sample of 53 of the 155 TI programs that transitioned in 2012. The transition readiness survey was administered just before transition, to the program manager of each TI and captured information about communication, capacity building and alignment. It employed structured questions supported by open-ended explanations. The survey also collected data on service utilization trends pre-transition. Because the evaluation was commissioned only in 2009, the transition readiness survey could not be conducted for the 2009 transition round.

An institutionalization survey was implemented approximately 9–12 months after transition. It sought to revisit all 80 of the TIs previously surveyed, however, due to merging of some TIs and late transition of others, only 70 TIs were finally included in the survey. The institutionalization survey, also administered to program managers and employing structured questions supported by open-ended explanations, assessed the extent to which key Avahan practices[14] had been institutionalized. The institutionalization survey also collected data on post-transition service utilization.

Fifteen case studies of TI programs were conducted approximately 6 months after transition, with 5 being revisited 1–3 years later. Case studies were selected to represent variation in state, key population served, and NGO versus CBO operations. Data collection comprised semi-structured interviews with TI managers, SLP staff overseeing the TI, SACS/TSU staff; and focus group discussions with outreach workers, peer educators and key populations. In total 83 semi-structured interviews, and 45 focus group discussions were conducted. Basic data on service provision and other relevant documents were collected from the TI. Case studies sought to understand the experience of transition and the effects of transition.

The study components described above were supplemented by regular interviews and discussions with the national and state level BMGF staff managing the transition. The research team also undertook a related piece of work that explored the role Avahan had played in influencing the Third National AIDS Control Program (NACP III) [15,16]. This study has helped inform the analysis of political commitment. Our assessment of political commitment to transition and the continuation of targeted prevention programs for HRGs focused on institutional commitment (including specifically expressions of support in formal policy documents) and budgetary commitments[17].

### Data Analysis

Table 1 summarizes the study questions and the sources of data and types of variables used to address each of the study questions. It should be noted that for the first transition tranche in 2009 no survey data were available. Accordingly for that particular tranche, our analysis draws only upon the case studies conducted.

**Table 1:** Data sources and type of analysis by Study Question.

	Data Sources & Type of Analysis		
Study question & dimension	Surveys: Transition readiness survey & Institutionalization survey	Case studies	Other
**Implementation of Transition**			
Content of Avahan transition strategy			Document review, Avahan documents, presentations & interviews with Avahan Staff
Implementation of Strategy		Interviews with SLPs, Avahan and TI Program managers	Interviews with Avahan Staff at national and state level; Additional study examining Avahan influence on development of India’s National AIDS Control Strategy IV (15).
**Transition readiness**			
Capacity development	Proportion of staff of TI programs receiving relevant training. (transition readiness survey)	Interviews with program staff, SLPs, SACS on strengths and weaknesses of TI	
Communication	Extent to which transition plans had been developed with input from HRGs. (transition readiness survey)	Interview with program staff, SLPs & SACS on the nature of relationships between stakeholders, and the form and frequency of communication regarding transition.	
Alignment: clinical & non-clinical	% TI program manager respondents reporting alignment with clinical norms (eg. STI syndromic management, referrals to ICTC) & non-clinical norms (eg. Procurement systems for drugs and condoms, staffing norms, and budgets.) (transition readiness survey)		
Political commitment			Institutional commitment expressed via government policy documents; Budgetary commitment expressed via budgets and expenditure reports
**Institutionalization**			
Management practices	Extent to which Avahan practices such as extensive use of data at different levels, strong onsite supervision, use of clinical and operational guidelines, flexible management, occurred post transition (institutionalization survey)	Interview data from TI Program managers, HRGs, and state level actors regarding changes to the program post-transition.	Delphi study used to identify key characteristics of Avahan programs that should be institutionalized post transition (14).
Uninterrupted funding & supplies	Extent to which Avahan practices such as on-time, adequate and uninterrupted flow of funds and commodities to TIs occurred post-transition (institutionalization survey)	As above	As above
Peer educator support	Extent to which Avahan practices such as rigorous performance based management of PEs, use of pictorial micro-planning tool,& needs based training for PEs continued post-transition (institutionalization survey)	As above	As above
Community mobilization	Extent to whichAvhan practices such as community-led crisis response, fostering of community groups, programs oversight by community members occurred post transition (institutionalization survey)	As above	As above
***Outcomes***			
Service Coverage	(i) Average % HRG population contacted by a Peer educator each month (ii) Average number of condoms distributed per HRG per month (extracted from health information system at TI Programs during institutionalization survey)		

For the transition readiness and institutionalization surveys, responses were translated into English where necessary, and entered into and analyzed in Excel. For measures of alignment three simple response categories were created reflecting full alignment, partial alignment, and no change from Avahan modalities. Analysis of both surveys sought to compare the degree of transition readiness, by round, by state and by population served.

For the case studies, interviews and focus group discussions, data collection was conducted in the local language, recorded, then transcribed and translated into English in India. Data were coded in Atlas.ti using a framework approach and analysis was confirmed between researchers in the U.S. and India. For each case study, a separate report was drafted, synthesizing findings from the data available.

Data were triangulated within and across study components and early results were used to inform later data collection. Results from the first round of data collection were shared and discussed with Avahan and local counterparts. It should be noted that one component of the Avahan transition strategy (namely support to government capacity at national and state level) was not addressed in this evaluation.

### Ethics Statement

The study was submitted to and exempted by the Internal Review Board of the Johns Hopkins Bloomberg School of Public Health. It was approved by the YR Gaitonde (YRG) Medical, Educational and Research Foundation, Centre for AIDS Research and Education Institutional Review Board in India. Respondents to all components of the study were asked for and provided written consent, with the exception of community participants in focus group discussions in the longitudinal case studies who, for reasons of limited literacy, provided oral consent that was recorded by the enumerator. These consent procedures were approved by the YRG Institutional Review Board.

## Results

### What was the transition strategy and how was it implemented?

There was very early recognition of the need to transition the Avahan program to local counterparts. In 2005, barely two years after its initiation, the Avahan technical panel and partners discussed the need to move Avahan towards a “truly sustainable” program. At the time this was considered mainly in terms of strengthening community owners and transferring funding and management responsibilities to government. This commitment to transition was formalized in a 2006 Memorandum of Cooperation with government[18]. A 2008 Avahan strategy document made a first attempt to lay out the transition strategy. This was formalized in a 2009 Memorandum of Cooperation that set out a clear transition timeline and transition responsibilities for government and Avahan[19].

The transition strategy continuously evolved responding to new learning as well as shifts in context, but key elements of the strategy included:

- Strengthening government systems at national and state level to manage TIs, through the provision of assistance to the Technical Support Unit at NACO, and through funding of state-level Technical Support Units.
- Building NGO/CBO capacity to run TI programs independently through training for NGO/CBO staff.
- Enhancing key populations’ capacity to demand and access services and information, and promote their engagement in building an enabling policy and program environment through community mobilization activities conducted by State Lead Partners at TI Program staff.
- Aligning clinical and non-clinical aspects of the Avahan program with government systems prior to handover. In terms of clinical alignment this involved new clinical protocols (such as for STI syndromic management) and referring clients to government health services such as Integrated Counseling and Treatment Centers (ICTC). Non-clinical dimensions included alignment with NACO norms for budgets, staffing profiles and procurement systems, as well as adoption of the government’s health management information system.
- Promoting continued government commitment to targeted prevention programs and transition of the Avahan program, through engagement with government in the development of key policies such as the National AIDS Control Program III.

The 2009 transition round was problematic. While upper and middle management levels at NGOs/CBOs running the TI programs and state-level organizations were relatively well informed about transition, frequently peer educators were less well informed, notified just prior to the transition or were unaware that transition was taking place. Transition preparation focused on budget and staffing alignment, but failed to anticipate operational challenges TIs would face post-transition, notably shortages of cash and commodities due to delays in initiating budget disbursements and supplies via government systems. While transition was meant to occur in April 2009, frequently government contracts to NGOs/CBOs were not issued until months later. There was no planning for the kind of post–transition support that SLPs might provide after transition, which sometimes resulted in confusion at the TI level with both SACS and SLPs intervening. In one state the SLP was asked by SACS to cease its involvement with TI programs that had transitioned, thus undermining any post-transition support.

Informants suggested that one reason for the challenges encountered in the 2009 transition round were the short time frame for planning transition. In our case studies we found efforts to prepare transitioning TI programs only started 1–3 months before transition. In addition a lack of experience in collaboration between Avahan and government sometimes manifested itself in mistrust.

By comparison, the 2011 transition round appeared better organized. Relationships between Avahan and government were stronger and communications between these actors were more effective and built on trust. New posts for “transition managers” were established within SLPs, as well as at the central level in BMGF, ensuring a clearly identified point-person with responsibility for the transition. Clear indicators to judge the level of program interventions, and budget lines to support transition were established. There was a longer period of transition preparation, with TIs starting preparation on average 6 months before transition according to the transition readiness survey. In two states, Karnataka and Maharashtra, buffer stocks of condoms, medicines and occasionally funds were built up to address potential gaps in supplies. Further, arrangements for post-transition support, which allowed SLPs to continue to work with TI programs, particularly on community mobilization activities were formally agreed in advance. These improvements were facilitated by the fact that the second transition round came a full two years after the initial round, was relatively small (comprising just 28 TIs), and therefore relatively straightforward to implement.

The 2012 transition round appeared to go smoothly despite its large size. This success built on even earlier preparation (starting on average 9 months before transition), ongoing strong collaborations and effective communication between SLPs, SACs and TSUs. Post-transition support packages, especially for community mobilization, were standardized across TIs. Buffers of funding and commodities were established for all TIs. Overall planning and implementation of transition strategies were guided by a Common Minimum Program for transition. Data from the surveys reinforce the positive impression that case study data provide of effective implementation of transition strategies in later rounds: while 79% of TI managers in the 2011 round agreed or strongly agreed that “the transition experience went smoothly”, 93% of TI managers perceived the last round of transition to have gone smoothly.

### How well prepared was the Avahan program for transition?

In terms of developing capacity and promoting engagement of TI staff and key populations, there was a gradual increase over time in the amount of staff training provided to help prepare for transition. For example, overall more field staff attended transition trainings in 2012 versus 2011 (60% of TIs vs. 15%, respectively), and more TI managers received training on changes to TI guidelines in 2012 compared 2011 (83% vs. 67%, respectively). However higher-level staff received more training than lower-level staff, and for the April 2012 transition most staff training for transition took place very late in the process.

In terms of alignment of clinical aspects of the program, Avahan TIs had previously delivered most clinical services for general health and sexually transmitted infections through TI-run centers or alternatively had contracts with private clinics for services. During transition, the delivery of clinical services shifted away from TIs to government facilities. Typically this also implied a shift from broad services for sexually transmitted infections, and indeed general health services, toward a narrower focus on integrated counseling and testing for HIV and syndromic treatment for STIs.

Overall, we found high levels of clinical alignment and very high referral rates to government ICTCs (Table 2). In a number of states, as part of the transition process, government sought to sensitize employees at government clinics so as to improve stigma and discrimination previously felt by clients at these facilities. These efforts appeared successful for FSW, but less so for MSM and transgendered populations, and also varied from one state to another.

**Table 2:** Measures of clinical and non-clinical alignment of Avahan programs with government prior to transition.

Percentage of TIs:	2011				2012			
	Andra Pradesh (n = 11)	Karnataka (n = 6)	Tamil Nadu (n = 4)	Maharashtra (n = 6)	Andra Pradesh (n = 16)	Karnataka (n = 17)	Tamil Nadu (n = 7)	Maharashtra (n = 13)
***Clinical Alignment***								
Following STI syndromic management guidelines of NACO	82%	100%	100%	67%	100%	71%	86%	85%
Referring most cases to government ICTC	100%	100%	100%	83%	100%	100%	58%	100%
***Non-clinical alignment***								
Following all SACS reporting formats	100%	100%	100%	83%	100%	100%	29%	100%
Following SACS TI team structure	100%	83%	100%	83%	100%	100%	100%	85%
Procuring STI syndromic management medicines as per NACO/SACS guidelines*	73%	17%	100%	33%	0%	59%	71%	38%
Procuring all condoms through channels suggested by SACS*	100%	33%	100%	17%	100%	71%	71%	46%
Following NACO/SACS budget guidelines	91%	100%	100%	83%	100%	100%	43%	77%

* Where relatively low rates of alignment with procurement channels are noted, respondents explained that buffer stocks had been established so that they had not started to use government systems for procurement.

Avahan also needed to align managerial and operational aspects of the program prior to transition. We found high alignment of budgets, TI team structure, and reporting and procurement systems (Table 2). Where procurement systems were not aligned, respondents explained that they had buffer stocks, and therefore had not started using government systems. Many TIs described cutting salaries in order to align with government norms, and some also cut staff—typically a difficult process. Often, alignment implied budget cuts for trainings, meetings, travel allowance, outreach, and office expenditure. Salaries for TI workers also declined, although there were a few exceptional circumstances where salaries increased or were protected. Sometimes SLPs managed to negotiate some flexibility around government norms from SACS so as to help protect health workers’ pay. New (higher level) literacy requirements also resulted in difficulties for staff recruitment and led to turnover, especially among key population members who had been recruited as NGO staff.

With respect to government commitment, Avahan was fortunate to be transitioning its program into the context of a highly committed and matured government program, whose policy goals aligned with Avahan HIV prevention objectives. The Third National AIDS Control Program (NACP III)[16] committed to quadrupling government funding for HIV/AIDS and set out a clearer focus than previously on HRGs. While Avahan was too newly established to influence NACP III policy formulation, it was substantially involved in the development of norms and guidelines for NACP III implementation[15]. Avahan staff invested significant energy, with considerable success, in working closely with NACO to develop norms and guidelines that built on Avahan learning. The government delivered on the substantially scaled up funding that was promised, providing adequate funding throughout the course of the program[20].

### How well institutionalized is the Avahan program?

A number of key practices that Avahan pursued were identified as critical to the success of the program[14]. These key practices can be grouped into four categories:-

- **Management practices,** notably extensive use of data at different levels of the system, strong onsite supervision, use of clinical and operational guidelines, a “common minimum package”, and flexible management;
- **Funding and supplies,** including on-time, adequate and uninterrupted flow of funds and commodities to TIs;
- **Peer-educator (PE) support,** comprising rigorous performance-based management of PEs, use of pictorial micro-planning tool, and needs based training for PEs.
- **Community engagement,** including program oversight by committees made up of community members, strong support to community groups, and community-led crisis-response mechanisms to respond to violence or harassment of community members.

The institutionalization survey assessed the persistence of these practices post-transition.

Overall, by the time of the institutionalization survey, 9–12 months after transition, there had been significant program changes across these dimensions. 75% of survey respondents from the 2011 transition round and 69% from the 2012 round agreed with the statement that “The overall program has changed significantly as compared to pre-transition.”

Over 50% of TI program managers noted changes in key management practices (Table 3). In general, these changes in management practices were viewed positively. For example, respondents noted improvements in supervisory structures, clarity of guidelines and use of data for planning and tracking progress. The one exception was in terms of flexibility in management. Across both rounds, 57% of TI program managers viewed the government system to be less flexible than Avahan, and were particularly concerned about the implications of limited financial flexibility such as ability to move money between budget lines or carry funds from one period to the next.

**Table 3:** Change in Avahan practices post transition.

	Has this key practice changed since transition? (YES)		Was this a change for the better? (YES)	
	2011 (n-28)	2012 (n = 42)	2011	2012
***Management***				
Supervision of your work by SACS/DAPCU/TSU	75%	60%	90%	76%
Clarity of guidelines on clinical services	43%	26%	92%	91%
Use of data for program planning	57%	62%	88%	88%
Use of data to monitor program progress	68%	60%	79%	88%
Flexible management style	54%	60%	0%	0%
***Adequate and uninterrupted flow of stocks and funds***				
Quantity of commodities supplied	29%	36%	75%	40%
Supply chain of commodities	61%	64%	71%	41%
Amount of funds	75%	60%	7%	0%
On time funds	43%	62%	0%	0%
***Peer educator supervision and support***				
The use of pictorial micro-planning	46%	40%	92%	82%
Performance of peer outreach workers monitored rigorously	50%	64%	93%	93%
Training for peer educators	64%	55%	56%	48%
***Community activities***				
Community-led crisis response management	36%	31%	80%	54%
Focus on supporting community groups	36%	26%	60%	73%
Oversight by committees of community members	36%	19%	90%	88%

With respect to funding and supplies, close to 70% of TI programs always had sufficient stocks of condoms and medicines, but the remaining 30% had experienced commodity stock outs within a few months post-transition largely due to changes in the supply source and schedules. In all, 10% of TIs regularly had problems with cash flows and less than half of the TIs *never* had cash flow problems. Further, the transition reduced budgets for 63% (across both rounds) of TIs, which in turn decreased TI staff salaries, travel allowance, and funding for TI events and activities.

In 59% of TIs (across both rounds), performance monitoring of *peer educators* was felt to have changed, largely for the better. There appeared to be closer monitoring of peer educators, with more concrete performance standards, and termination of contracts for those who did not meet standards. Further, 59% of TIs (across both rounds) also thought that peer educator training had changed, though views were mixed on whether this had led to improvement. Due to more restricted budgets, the frequency of training had declined, but training opportunities may have been better targeted than previously. The specific use of a pictorial microplanning tool to map HRGs had also changed for 43% of TIs, which was largely perceived as positive.

Based upon the institutionalization survey, about one third of TIs experienced changes in community engagement across the three activities considered. The majority of TI program managers viewed these to be changes for the better; however, a different perspective emerged from community members who participated in the focus group discussions conducted as part of the longitudinal case studies. Community members perceived that TI programs had shifted away from providing incentives and community recognition activities towards a more narrowly clinical focus. Budget cuts that removed small amounts of funding for tea and refreshments for community members had disproportionately large effects on the motivation and satisfaction of community members. Shifts in the source of clinical services also resulted in mixed feelings: while many community members appreciated the possibility of being mainstreamed into government clinics, and recognized improvements in government provider sensitivity to their needs, they also mourned the loss of general health services at the TI. Many resented the focus on testing to the exclusion of other activities. MSM and transgender communities appeared particularly adversely affected by the shift.

### Were program outcomes sustained after transition?

**Fig 3:**
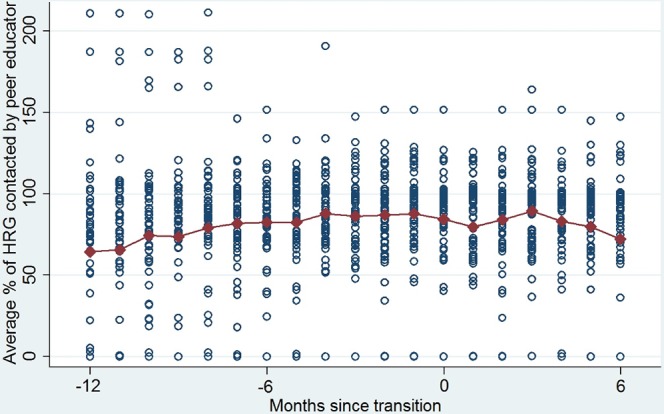
Sustained outcomes: High risk groups contacted by peer educators.

**Fig 4:**
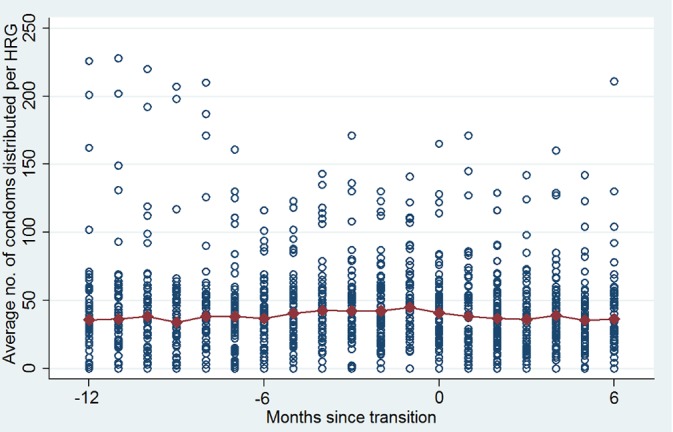
Sustained outcomes: Condom distribution to members of high risk groups.

Figs 3 and 4 show trends in services before and after transition in a pooled sample of TIs that transitioned in 2011 and 2012. Service coverage is captured by the average percentage of HRGs contacted by peer educators per month (Fig 3) and the average number of condoms distributed per HRG per month (Fig 4). Data were standardized around the same transition date, and pooled as there was no clear difference in trends in services between TI programs transitioning in 2011 and 2012.Overall, it appears that service coverage was sustained at least up to six months post transition.

## Discussion

While questions of transition and country ownership are of growing importance across a number of major development partners, we are not aware of other prospective evaluations of program transitions. This transition assessment was structured around five questions. Here we summarize our conclusions concerning the first four questions, based upon the evidence presented above. We then address the fifth question: “What are the elements necessary for effective transition of HIV/AIDS prevention programs?”, before describing limitations of the study.

With the exception of the first small 2009 round of transition, the Avahan transition appeared to be well implemented. The phased nature of the transition allowed for learning between each tranche, and preparations for and investments in transition increased in scope and complexity over time. Transition preparations were varied, encompassing measures to promote continued high level government commitment to HIV/AIDS prevention activities; adaptations to programs to support budgetary, operational and clinical alignment; and support to the development of capacities among key stakeholders. Our assessment identified relatively high levels of transition readiness across these dimensions. Both the 2011 and 2012 transition rounds occurred without significant disruptions to services, where effective transition preparation stands out as an important explanatory factor.

In terms of institutionalization, we found very significant changes to management practices 9–12 months after transition. Many of the adaptations in the program were perceived to be positive ones by TI managers with the clear exceptions of more limited funding, and less flexibility in program management. Transitioning a donor-run program into a government health system will likely create adaptations in the government system as well as compel adaptation in the program itself. This begs the question as to which adaptations are an acceptable, or even necessary part of transition, and which are likely to damage the long-run sustainability of program outcomes.

While TI program managers had positive impressions of program transition, the views of community members were much less so and the community mobilization component of the program suffered most as a consequence of transition. This was partially addressed by BMGF through the establishment of 12 month post-transition support agreements between SLPs and the TI programs that provided some ongoing support, particularly to community mobilization. BMGF provided three further years of limited support for community strengthening focusing on specific elements such as fund-raising, but the long-term future of community mobilization is unclear.

Finally, our data suggest that core services (condom distribution and peer counseling) provided by TI programs continued at similar coverage levels post transition.

### Elements important for an effective transition

Many of the lessons from this evaluation echo those of the program implementers[9]. We identify five elements of the transition design and implementation that contributed to a successful transition.

1. **Extended timeline and phased transition—**The extended (2009–2013) timeline allowed for substantial transition preparation. Further, the phased nature of the transition facilitated learning and feedback based on prior transition rounds. We observed increasing investment in and a more standardized approach to transition preparation, as both Avahan and government actors learned what was necessary to support transitioning TI programs and acted upon this learning in later transition rounds. The extended timeline also supported the development of trust between stakeholders and open lines of communication.
2. **Substantial investment in transition preparation**–Avahan made a major investment in preparing the program for transition, particularly focused on aligning the program, ensuring clear communications and agreements with local stakeholders, and enhancing capacity of local partners to manage the program. Specific transition management strategies emerged after the 2009 transition round and were systematized in a Common Minimum Package for transition ahead of the 2012 transition round.
3. **Commitment to transition**–Avahan was fortunate to transition to a government that already had strong policy commitments to HIV/AIDS prevention that aligned with Avahan’s own goals. Moreover, the Government of India was prepared and willing to take over the funding and operation of Avahan services. However, this was not purely luck–Avahan sought to shape the policy environment to ensure strong commitment to prevention among HRGs by actively engaging in the development of NACP III. It also signaled a strong commitment to transition, through the memoranda of cooperation that helped convince all actors of the seriousness of transition plans.
4. **Communication and trust**–communications between key partners at state level (SLPs, SACS, and TSUs), as well those running TI programs were relatively strong and improved over time. Initially, during the 2009 transition round, state level actors demonstrated mutual suspicion, but the need to work together in the transition process meant that gradually trust between actors grew and highly collaborative working relationships were established. Specific activities to build communication and trust between those transitioning the program and those receiving it may be beneficial.
5. **Adaptability and willingness to provide additional support in key areas**—the initial Avahan transition strategy did not address community mobilization differently from other program elements. However, it became apparent during the transition process that government was less well placed, both in terms of orientation and expertise, to support community mobilization activities than the Avahan program. Avahan demonstrated flexibility by seeking to address this issue which was not part of the original plan. The post-transition support agreements, which had a strong focus on community mobilization helped mitigate, although perhaps only temporarily, some of the negative consequences of transition for community mobilization.

### Limitations

There are several limitations to the evaluation. Taking a broad perspective, this evaluation had an adequacy design[21], and we are therefore unable to causally link the transition preparations made to the smoothness of transition, or sustained program coverage. Instead our findings *suggest* that a high degree of transition preparedness contributes to smooth transition and the sustainability of program coverage.

There were a number of specific design weaknesses in the evaluation. First, while we assessed the amount of training provided, trainings do not necessarily translate into higher capacity, and we have no data on the impact of training on skills. Second, there was limited buy-in to the evaluation from the Government of India and this restricted opportunities for checking interpretation of data with government stakeholders and made it difficult to secure national or state-level data on TI service coverage post-transition. Thus, we were not able to compare the data collected from the transitioning Avahan TI programs with the broader population of TIs. Third, the evaluation did not measure the extent to which key Avahan practices were conducted prior to transition and instead relied on TI manager recall of how things had changed. Fourth, this evaluation collected data from TIs immediately before transition and approximately one year post-transition; however, many program changes were still transpiring at this time, and the Indian health system is also changing rapidly, thus the true long run effects of transition may still be emerging.

Finally, some features of the Avahan context including the high-level government policy commitment to HIV/AIDS prevention, the availability of significant government funding to support this commitment and relatively high government capacity (despite frequent challenges of high staff turnover) may limit transferability to other contexts.

## Conclusions

Transitioning a donor-funded and managed program to a country government frequently entails substantive changes in financing, management and clinical services. There is clearly a risk that such transitions disrupt services and undermine program sustainability[22]. The experience of the Avahan transition suggests that there is also potential for transition to be a largely positive, and enabling process, that both improves program functioning and enhances local leadership. Investing in advance in planning transitions and communicating these plans to all concerned stakeholders appears essential.

Multiple donors are currently considering how best to transition or graduate health programs, particularly in middle-income countries. There are very few evaluations similar to the one described here, and our understanding of the factors supporting effective program transitions is still nascent. Further monitoring and evaluation of transitions should be conducted to inform future strategy.

## Supporting Information

S1 Table Transition readiness survey data by state. (PDF)

S2 Table Institutionalization survey Round 1, 2011 Transition tranche. (PDF)

S3 Table Institutionalization survey Round 2, 2012 Transition tranche. (PDF)
