# Supplementary material for: Transitioning a Large Scale HIV/AIDS Prevention Program to Local Stakeholders: Findings from the Avahan Transition Evaluation
Source: PLoS One. 2015 Sep 1;10(9):e0136177. doi: 10.1371/journal.pone.0136177 (PMC4556643; doi:10.1371/journal.pone.0136177)
Supplement: S1 Table — (PDF) [file pone.0136177.s001.pdf]

Transition Readiness Dashboard

From Round 1 & 2 Transition Readiness Assessment 2011 & 2012

Evaluative Assessment of Phase II of the Avahan Program: Transition and Influence Goals

Introduction

This project assessed the transition readiness of Targeted Interventions (TIs) for high risk groups (HRGs) that are transitioning from Avahan management and funding to that of State AIDS Control Societies (SACS). This study component focused on how aligned Avahan TIs are prior to transition and how well prepared Avahan-funded Non-Government Organizations (NGOs) / Community-Based Organizations (CBOs) are for the transition. Round 1 of the transition readiness survey was conducted between April-June 2011 among all TIs that transitioned in Spring 2011 (n=27). Round 2 was conducted between April-June 2012 among a sample of TIs that transitioned in Spring 2012. Project Directors (PD) or Coordinators (PC) of 53 HRGs TIs participated in Round 2 of data collection across 4 states.

Table of Contents

This package includes the following:

Findings **1. Overall Results**

2. By State - Andra Pradesh

3. By State - Karnataka

4. By State - Tamil Nadu

5. By State - Maharashtra

6. Buffer & Flexibility by State

7. By NGO/CBO

8. By HRG Category

9. By Split TI

10. By Urban/Rural/Both

11. By April/Later Transition

Appendices **12. Explanations of Low/Medium/High Categories**

Transition Readiness Dashboard

From Round 1 & 2 Transition Readiness Assessment 2011 & 2012

Evaluative Assessment of Phase II of the Avahan Program: Transition and Influence Goals

1. Overall results across all HRGs

	HRG TIs Overall	
	2012	2011
No of TIs	53	27

	2012		2011	
	NGO	CBO	NGO	CBO
Are you a NGO or CBO?	34	19	17	10

	2012				2011			
	AP*	KR	TN	MH	AP*	KR	TN	MH
In which state are you located?	16	17	7	13	11	6	4	6

*AP: Andra Pradesh, KR: Karnataka, TN: Tamil Nadu, MH: Maharashtra

	2012			2011		
	FSW	MSM	Both	FSW	MSM	Both
For whom do you provide services?	20	11	22	14	2	11

	2012		2011	
	Not Split	Split	Not Split	Split
Did the TI Split due to transition?	26	27	25	2

	2012			2011		
	Urban	Rural	Both	Urban	Rural	Both
Is the TI located in an urban area, rural area or both?	28	11	14	16	4	7

	2012	
	April	Later
When did the TI transition?	43	10

Section 1: NGO/CBO Capacity

	2012			2011		
	None	Societies Registration Act	States Trusts Acts	None	Societies Registration Act	States Trusts Acts
Q 1. What is the legal status of the organization? (mention the registration details under the societies / public trusts as applicable)	0%	81%[A]	19%	0%	85%	15%

[A] *Among 27 TIs of 2012 transition that mentioned the year of registration, 7 were registered in the 1980s, 9 in the 1990s and 11 between 2000-2010.*

	2012		2011	
	No	Yes	No	Yes
Q 1.1. Does the organization have a Foreign Contribution (Regulation) Act 1976 account?	21%	79%[B]	37%	63%

[B] *Among 13 TIs of 2012 transition that mentioned having FCRA, 2 were registered in the 1980s, 1 in the 1990s and 10 between 2000-2011.*

	2012			2011		
	Low**	Medium	High	Low**	Medium	High
Q 2. Have the staff been informed about the transition?	0%	30%[A]	70%[B]	0%	26%	74%
Q 3. Does the NGO/CBO have a JAT (Joint Appraisal Team) score?	32%[C]	40%[D]	28%[D]	19%	37%	44%
Q 4. Has there been communication about empanelment from SACS?	15%	15%	70%	0%	26%	74%
Q 5. Has there been any change in the reporting format, and are you sending any reports to SACS/District AIDS Control Societies?	8%	2%	89%	0%	4%	96%
Q 6. Has there been any change in the TI team structure, and are you following the SACS/NACO guidelines?	0%	4%	96%	0%	7%	93%
Q 9. Is the NGO/CBO following the STI syndromic management guideline of NACO?	2%	13%[E]	85%	15%	0%	85%
Q 10. Does the NGO/CBO procure STI syndromic management medicines as per NACO/SACS guidelines?	53%[F]	9%	38%	37%[F]	7%	56%
Q 11. Has there been any change in the condom procurement process?	15%[G]	11%	74%	26%[G]	7%	67%
Q 12. Has there been any change in the budget as per NACO/SACS guidelines?	2%	11%[H]	87%[H]	0%	7%	93%

** See Part 2 Explanations for all cutoffs for Low/Medium/High; High means highly aligned

[A] *TIs reported being informed between April and December 2011, and the majority had meetings to discuss the transition in March 2012.*

[B] *TIs reported being informed between 2009-2010 (4 TIs), 2011 (19 TIs) and 2012 (4 TIs); the majority had meetings in March 2012.*

[C] *At least 3 TIs have not heard about JAT; 2 said they were exempt from JAT.*

[D] *More than 6 TIs indicated not knowing the JAT score. Among TIs that noted JAT timing, most took place between November-December 2011.*

[E] *Some TIs received 3, 5, 6 or 10 kits rather than 7 kits. Some MSM TIs noted that only 3 kits (Red, White, Gray) are relevant for MSM. A few TIs noted that kit availablity, it will some kits will be available one time and the same willnot be available of another time.*

[F] *Many of these TIs indicated that Avahan supply chain is still in place for medicines because they have a buffer stock in hand.*
Among 2012 TIs, 19 TIs indicated having a buffer stock, with the majority having a buffer stock for 6 months.

[G] *Some TIs indicated having a buffer stock of condoms in hand. Among 2012 TIs, 3 TIs indicated having a buffer stock.*

[H] *Many TIs described cutting salaries, and some also cut staff. For a few TIs, ORW salaries increased (mostly in AP). Some TIs noted budget cuts for trainings, meetings, travel allowance, outreach, office expenditure and increased frequency of payments. Some TIs described staff being underpaid for the hard work, not being happy with the salary, and in some cases leaving the TI.*

2012			2011		
Low	Medium	High	Low	Medium	High

Q 7. What is the present ratio of peer educators to HRG?*	0%	8%[A]	92%	0%	4%	96%
Q 8. What is the present ratio of outreach worker to HRG?**	0%	9%[B]	91%	4%	7%	89%

*NACO norm: Ratio of peer educators to HRG is 1: 60

**NACO norm: Ratio of outreach worker to HRG is 1: 250

[A] ***4 TIs (all in KR) reported having a ratio of peer educators to HRG of 1:120, where 1 peer educator is hired in place for 2 due to budget constraints.***

[B] ***Ratio of outreach worker to HRG ranged from 1:180 to 1:300.***

Q 13 & 14. Have the following staffs received training for the transition OR meetings on the transition as recommended by SACS?	2012 Trainings [A]			2012 Meetings [B]			2011 Trainings & Meetings		
Training for Transition, by Topic* **	None	1 staff	>1 staff	None	1 staff	>1 staff	None	1 staff	>1 staff
TI guidelines	9%	17%	74%	34%	4%	62%	0%	64%	36%
Program management	9%	21%	70%	36%	8%	57%	0%	45%	55%
Outreach planning	15%	15%	70%	43%	2%	55%	9%	9%	82%
Condom programing	19%	11%	70%	43%	2%	55%	36%	27%	36%
Community mobilization	26%	6%	68%	43%	2%	55%	27%	0%	73%
Communication	23%	13%	64%	47%	2%	51%	18%	18%	64%
Counselling	30%	8%	62%	45%	6%	49%	9%	36%	55%
STI management	21%	4%	75%	43%	2%	55%	0%	55%	45%

* Percentages indicate the proportion of TIs in the state where 0, 1 or more than 1 type of staff attended the training for transition.

** Percentages show training that has been received. It does not count training that has been planned but has not yet taken place.

[A] ***Some TIs noted not having any training specific to the transition or receiving training after the transition.***

[B] ***Some TIs (especially in KR) noted dissussing the transition during monthly meetings.***

Q 13 & 14. Have the following staffs received training for the transition OR meetings on the transition as recommended by SACS?	2012 Trainings			2012 Meetings			2011 Trainings & Meetings		
Training for Transition, by Staff*	Lowest	Average	Highest	Lowest	Average	Highest	Lowest	Average	Highest
PC/PD	0	6	12	0	4	8	2	5	8
Field Staff	0	4	12	0	4	8	0	4	8
Counsellor/ANM	0	4	12	0	4	8	0	2	5
Doctor	0	2	12	0	2	8	0	1	2
MIS Officer	0	4	12	0	3	8	0	2	8
ORW	0	5	12	0	4	9	0	0	0

* This indicates the lowest, average and highest numbers of types of trainings attended by each staff by state. Greater numbers indicate that staff in the respective category attended multiple types of trainings for the transition

	2012	
Q 15. Does the NGO/CBO have a buffer in any of the following areas?	No	Yes
Medicines	38%[A]	62%[B]
Condoms	60%[A]	40%[C]
Funds	75%[A]	25%[D]

[A] *Some TIs reported not having a buffer in all areas (especially in KR). Advanced planning often helped in these TIs where items were received 3 months in advance. 1 TI noted shortage of medicines without a buffer. Another TI noted taking from a trust when funds run out.*

[B] *TIs reported having a medicine buffer ranging from 2% to 1, 3, and 6 months. The majority (17 TIs) reported having a medicine buffer of 6 months.*

[C] *Some TIs reported having a condom buffer ranging from 5% or 60,000 condoms to 1, 3, and 6 months. 3 TIs reported having a buffer of 6 months.*

[D] *Few TIs reported having buffer for funds. Release of funds ranged by TI, from yearly, every 6 months, quarterly, to every 3 months.*

	2012					
	No flexibility		Flexibility provided		Total	
Q 16. Has there been any flexibility provided from NACO/SACS in the following areas?	Not important	Important	Not important	Important	Flexibility provided	Flexibility Important
Reporting	38%	49%	2%	11%	13%	60%
Staffing	17%	60%[A]	4%	19%[B]	23%	79%[C]
Budgets	13%	74%[A]	0%	13%	13%	87%[C]
STI services	36%	34%	4%	26%	30%	60%
Condom programming	45%	32%	2%	21%	23%	53%
Linkages to ICTC	30%	34%	0%	36%	36%	70%

[A] *Some TIs reported being compelled to follow all norms strictly. Some noted that SLPs used to help a lot; now KPs have to manage on their own.*

[B] *Some TIs (especially in TN) reported asking SACS for flexibility about providing more counsellors, where flexibility was granted.*

[C] *Many TIs reported flexibility to be important especially for staffing and budgets.*

Section 2: TI Alignment - From Interviews

	2012			2011		
	Low	Medium	High	Low	Medium	High
Q 1. Has there been any change in the Avahan method of micro-planning	21%[A]	17%[B]	62%[B]	19%	0%	81%
Q 2. Do all identified hotspots/halting places have condom outlets (TO, NTO, CVM)?	21%[C]	68%[D]	11%[E]	0%	19%	81%

[A] *Some TIs reported that SACS had not informed about micro-plans, SACS does not have a micri-planning tool, or that they have not had any recent training by SLP/SACS. 1 TI noted that they are not using micro-planning.*

[B] *Some TIs reported receiving training by SLP/SACS on micro-plans. 1 TI noted that 17 NACO formats were added. Another TI noted that SACS micro-plan methods are old and that TIs need social network based planning. Respondents listed between 2 to 7 microplans they use, which are conducted every 3, 4, or 6 months. 1 TI noted using microplans for one-year planning.*

[C] *11 TIs reported removing all condom outlet boxes and only using direct hand to hand distribution of condoms. Outlets were closed because it was not used properly or KPs had asked not to put outlets. Some described distributing through PE, outreach, pan shop, or in a 'condom meeting.'*

[D] *Many TIs reported using a combination of outlets and direct distribution of condoms. Among this group, on average 27% of hotspots had outlets. Outlets were not used in some areas because people throw them away, or because the 'police or public creates trouble.' The majority of direct distribution was through PE and outreach. Other methods included distributing at community houses, leaders' houses, PE's house, homes of FSW, STI clinic, lodges, NTO, CVM, and hidden in known bushes.*

[E] *Some TIs reported also creating outlets on a temporary basis during festivities, or using social marketing.*

	2012		2011	
Q 3. Does the NGO/CBO have a linkage with the following government services?	Most cases are referred	Most cases referred receive these services*	Most cases are referred	Most cases referred receive these services*
Government ICTC	94%	83%[A]	96%	33%
ART centres	100%	81%	100%	30%
TB screening centres	96%	81%[B]	100%	37%

* Percentages show NGO/CBOs where most cases are referred to government services AND those referred receive these services

[A] ***Some TIs reported good links with ICTC with rapport and support, or having linkage established for 1-8 years. Some TIs refer to PPP centers. 1 TI noted: 'We are facing problems from government ICTC,as soon as they hear our organisation name.'***

[B] ***Some TIs reported giving attention to TB screening, while 1 TI reported "Government TB screening timing is not suited for us."***

	2012			2011		
Q 4. Which places do people go for STI services?	1st Choice (most preferred)	2nd Choice	3rd Choice	1st Choice (most preferred)	2nd Choice	3rd Choice
On-site clinic, Part time	21%[A]	4%	8%	30%	4%	0%
On-site clinic, Full time	47%[A]	6%	25%	56%	7%	4%
Government hospital clinic	8%[B]	72%	17%	11%	56%	26%
Preferred private practitioner	25%[C]	19%	51%	4%	33%	56%
None	0%	0%	0%	0%	0%	14%

[A] ***Some TIs reported that people prefer on-site clinic because they provide good services, counselling, confidentiality, privacy, free treatment, flexible timing, and are trusted.***

[B] ***Some TIs reported that the government clinic time is restictive, or that government doctors do not treat the transgenders properly and that the general community teases them at government hospitals.***

[C] ***Some TIs reported using private providers if they can pay, because they do not have to take time out and does not discriminate.***

	2012			2011		
	Low	Medium	High	Low	Medium	High
Q 5. Has the NGO/CBO been able to form groups at the community level?	2%[A]	9%[A]	89%[B]	0%	0%	100%
Q 6. Have community members at the hotspots formed crisis response committees?	4%[C]	13%[C]	83%[D]	0%	15%	85%

[A] ***Some TIs reported that groups were trying to form, not recognized or not registered. 1 TI noted a CBO that was formed that is not actively working. Another TI noted "they can form groups but difficult to follow."***

[B] ***Some TIs reported groups with up to 6 committees having monthly meetings.***

[C] ***Some TIs reported that nobody is ready to take up the responsibility, or that there is a budget problem with no allowance for meetings so meetings only take place once in 3-4 months.***

[D] ***Many TIs reported having functional crisis committes that meet every month to discuss problems about police, partners, boundary, clients, auto drivers, rowdiness, clinic, and neighbor. Some TI noted responding to a crisis such as a raid in a brothel or intervening when FSWs are stabbed or injured. 1 TI noted having lawyers who can also help. 1 TI noted that 'people trust us and work with us.'***

Section 3: TI Alignment - From CMIS Avahan

	2012						
	Low	Medium	High	Don't know / NA	Data not available	# TIs with data	Average value
Q 1. What is the coverage of identified HRGs with regular contact (two contacts each month)?	0%*	4%	96%	0%	0%	53	93%
Q 2. What is the coverage of free condom supply among the identified HRG by NGO/CBO?	0%	15%	30%	55%****	51 (96%)	2	-
Q 3. What percentage of HRGs who are referred actually visit the ICTC?	43%**	17%	40%	0%	0%	39**	77%
Q 4. What is the coverage of syndromic management for HRGs with STI?	23%***	17%	60%	0%	0%	43***	108%

* Percentages show breakdowns among available data
** Includes 14 Tis where no referrals were made
*** Includes 10 TIs where no STI diagnosis were made
**** Not following Avahan format for condom supply

	2011						
	Low	Medium	High	Don't know	Data not available	# TIs with data	Average value
Q 1. What is the coverage of identified HRGs with regular contact (two contacts each month)?*	4%	4%	92%	0%	1 (4%)	26	89%
Q 2. What is the coverage of free condom supply among the identified HRG by NGO/CBO?**	0%	30%	70%	0%	7 (26%)	20	122%
Q 3. What percentage of HRGs who are referred actually visit the ICTC?***	10%	33%	57%	0%	6 (22%)	21	94%
Q 4. What is the coverage of syndromic management for HRGs with STI? ****	5%	23%	72%	0%	4 (15%)	23	110%

*NACO norm: 60% of identified HRGs have at least two contacts each month
**NACO norm: 100% of identified HRGs have access to free condoms
***NACO norm: 100% of HRGs who are referred should visit the ICTC
****NACO norm: 100% of HRGs with STI syndromes receive treatment

Transition Readiness Dashboard

From Round 1 & 2 Transition Readiness Assessment 2011 & 2012

Evaluative Assessment of Phase II of the Avahan Program: Transition and Influence Goals

2. Results of HRGs in ANDRA PRADESH

	AP 2012	AP 2011
Number of TIs interviewed in state	16	11

	AP 2012		AP 2011	
	NGO	CBO	NGO	CBO
Are you a NGO or CBO?	14	2	9	2

Section 1: NGO/CBO Capacity

	AP 2012			AP 2011		
	None	Societies Registration Act	States Trusts Acts	None	Societies Registration Act	States Trusts Acts
Q 1. What is the legal status of the organization? (mention the registration details under the societies / public trusts as applicable)	0%	100%	0%	0%	100%	0%

	AP 2012		AP 2011	
	No	Yes	No	Yes
Q 1.1. Does the organization have a Foreign Contribution (Regulation) Act 1976 account?	13%	88%	0%	100%

	AP 2012			AP 2011		
	Low*	Medium	High	Low*	Medium	High
Q 2. Have the staff been informed about the transition?	0%	31%	69%	0%	0%	100%
Q 3. Does the NGO/CBO have a JAT (Joint Appraisal Team) score?	0%	50%	50%	0%	9%	91%
Q 4. Has there been communication about empanelment from SACS?	0%	13%	88%	0%	0%	100%
Q 5. Has there been any change in the reporting format, and are you sending any reports to SACS/District AIDS Control Societies?	0%	0%	100%	0%	0%	100%
Q 6. Has there been any change in the TI team structure, and are you following the SACS/NACO guidelines?	0%	0%	100%	0%	0%	100%
Q 9. Is the NGO/CBO following the STI syndromic management guideline of NACO?	0%	0%	100%	18%[1]	0%	82%
Q 10. Does the NGO/CBO procure STI syndromic management medicines as per NACO/SACS guidelines?	100%[2]	0%	0%	27%[2]	0%	73%
Q 11. Has there been any change in the condom procurement process?	0%	0%	100%	0%	0%	100%

Q 12. Has there been any change in the budget as per NACO/SACS guidelines?	0%	0%	100%	0%	9%	91%

* See Section on Explanations for all cutoffs for Low/Medium/High; High means highly aligned

[1] NGO has a stock of Avahan STI kits (11 kits), which they have been instructed to use up before they switch in May 2011.

[2] Avahan supply chain is still in place; for some TIs there was buffer stock created by SLP to stock STI medicine.

	AP 2012			AP 2011		
	Low	Medium	High	Low	Medium	High
Q 7. What is the present ratio of peer educators to HRG?*	0%	0%	100%	0%	0%	100%
Q 8. What is the present ratio of outreach worker to HRG?**	0%	6%	94%	0%	0%	100%

*NACO norm: Ratio of peer educators to HRG is 1: 60

**NACO norm: Ratio of outreach worker to HRG is 1: 250

Q 13 & 14. Have the following staffs received training for the transition OR meetings on the transition as recommended by SACS?	AP 2012 Trainings			AP 2012 Meetings			AP 2011 Trainings & Meetings		
Training for Transition, by Topic* **	None	1 staff	>1 staff	None	1 staff	>1 staff	None	1 staff	>1 staff
TI guidelines	25%	56%	19%	81%	13%	6%	0%	64%	36%
Program management	25%	50%	25%	81%	13%	6%	0%	45%	55%
Outreach planning	31%	50%	19%	88%	6%	6%	9%	9%	82%
Condom programing	44%	38%	19%	88%	6%	6%	36%	27%	36%
Community mobilization	63%	13%	25%	94%	0%	6%	27%	0%	73%
Communication	50%	38%	13%	94%	0%	6%	18%	18%	64%
Counselling	88%	6%	6%	94%	0%	6%	9%	36%	55%
STI management	63%	13%	25%	88%	6%	6%	0%	55%	45%

* Percentages indicate the proportion of TIs in the state where 0, 1 or more than 1 type of staff attended the training for transition.

** Percentages show training that has been received. It does not count training that has been planned but has not yet taken place.

Q 13 & 14. Have the following staffs received training for the transition OR meetings on the transition as recommended by SACS?	AP 2012 Trainings			AP 2012 Meetings			AP 2011 Trainings & Meetings		
Training for Transition, by Staff*	Lowest	Average	Highest	Lowest	Average	Highest	Lowest	Average	Highest
PC/PD	0	4	7	0	1	5	2	5	8
Field Staff	0	1	4	0	0	4	0	4	8
Counsellor/ANM	0	1	6	0	0	4	0	2	5
Doctor	0	0	2	0	0	4	0	1	2
MIS Officer	0	1	4	0	0	4	0	2	8
ORW	0	1	6	0	0	4	0	0	0

* This indicates the lowest, average and highest numbers of types of trainings attended by each staff by state. Greater numbers indicate that staff in the respective category attended multiple types of trainings for the transition

	AP 2012	
Q 15. Does the NGO/CBO have a buffer in any of the following areas?	No	Yes

Medicines	6%	94%
Condoms	88%	13%
Funds	100%	0%

	AP 2012			
	No flexibility		Flexibility provided	
Q 16. Has there been any flexibility provided from NACO/SACS in the following areas?	Not important	Important	Not important	Important
Reporting	69%	31%	0%	0%
Staffing	31%	69%	0%	0%
Budgets	6%	94%	0%	0%
STI services	88%	13%	0%	0%
Condom programming	94%	6%	0%	0%
Linkages to ICTC	75%	25%	0%	0%

Section 2: TI Alignment - From Interviews

	AP 2012			AP 2011		
	Low	Medium	High	Low	Medium	High
Q 1. Has there been any change in the Avahan method of micro-planning	0%	13%	88%	0%	0%	100%
Q 2. Do all identified hotspots/halting places have condom outlets (TO, NTO, CVM)?	0%	100%	0%	0%	0%	100%

	AP 2012		AP 2011	
Q 3. Does the NGO/CBO have a linkage with the following government services?	Most cases are referred	Most cases referred receive these services*	Most cases are referred	Most cases referred receive these services*
Government ICTC	100%	100%	100%	0%[3]
ART centres	100%	100%	100%	0%[3]
TB screening centres	100%	100%	100%	0%[3]

* Percentages show NGO/CBOs where most cases are referred to government services AND those referred receive these services

[3] Across all TIs, PC/PDs did not perceive that most people who are referred actually receive the services.

	AP 2012			AP 2011		
Q 4. Which places do people go for STI services?	1st Choice (most preferred)	2nd Choice	3rd Choice	1st Choice (most preferred)	2nd Choice	3rd Choice
On-site clinic, Part time	25%	0%	0%	9%	0%	0%
On-site clinic, Full time	75%	0%	13%	82%[4]	0%	0%
Government hospital clinic	0%	94%	6%	0%	55%	45%
Preferred private practitioner	0%	6%	81%	9%	45%	45%
None	0%	0%	0%	0%	0%	10%

[4] PC/PDs discussed that people prefer the on-site clinic because it "has good lady doctor"; maintains confidentiality, is free of cost, does not discriminate, offer counseling, and "has good reputation."

	AP 2012			AP 2011		
	Low	Medium	High	Low	Medium	High
Q 5. Has the NGO/CBO been able to form groups at the community level?	0%	6%	94%	0%	0%	100%
Q 6. Have community members at the hotspots formed crisis response committees?	0%	0%	100%	0%	0%	100%

Section 3: TI Alignment - From CMIS Avahan

	AP 2012						
	Low	Medium	High	Don't know / NA	Data not available	# TIs with data	Average value
Q 1. What is the coverage of identified HRGs with regular contact (two contacts each month)?	0%	0%	100%	0%	0%	16	95%
Q 2. What is the coverage of free condom supply among the identified HRG by NGO/CBO?	0%	25%	63%	13%	15 (94%)	1	-
Q 3. What percentage of HRGs who are referred actually visit the ICTC?	81%	6%	13%	0%	9 (56%)	9	42%
Q 4. What is the coverage of syndromic management for HRGs with STI?	6%	19%	75%	0%	1 (6%)	15	118%

	AP 2011						
	Low	Medium	High	Don't know	Data not available	# TIs with data	Average value
Q 1. What is the coverage of identified HRGs with regular contact (two contacts each month)?*	0%	0%	100%	0%	1 (9%)	10	90%
Q 2. What is the coverage of free condom supply among the identified HRG by NGO/CBO?**	0%	20%	80%	0%	1 (9%)	10	141%
Q 3. What percentage of HRGs who are referred actually visit the ICTC?***	17%	33%	50%	0%	5 (45%)	6	83%
Q 4. What is the coverage of syndromic management for HRGs with STI? ****	20%	0%	80%	0%	1 (9%)	10	99%

*NACO norm: 60% of identified HRGs have at least two contacts each month
**NACO norm: 100% of identified HRGs have access to free condoms
***NACO norm: 100% of HRGs who are referred should visit the ICTC
****NACO norm: 100% of HRGs with STI syndromes receive treatment

Transition Readiness Dashboard

From Round 1 & 2 Transition Readiness Assessment 2011 & 2012

Evaluative Assessment of Phase II of the Avahan Program: Transition and Influence Goals

3. Results of HRGs in KARNATAKA

	KR 2012	KR 2011
Number of TIs interviewed in state	17	6

	KR 2012		KR 2011	
	NGO	CBO	NGO	CBO
Are you a NGO or CBO?	7	10	6	0

Section 1: NGO/CBO Capacity

	KR 2012			KR 2011		
	None	Societies Registration Act	States Trusts Acts	None	Societies Registration Act	States Trusts Acts
Q 1. What is the legal status of the organization? (mention the registration details under the societies / public trusts as applicable)	0%	100%	0%	0%	100%	0%

	KR 2012		KR 2011	
	No	Yes	No	Yes
Q 1.1. Does the organization have a Foreign Contribution (Regulation) Act 1976 account?	18%	82%	100%	0%[1]

[1] Some TIs have applied, while others have not yet done so but would like to apply

	KR 2012			KR 2011		
	Low*	Medium	High	Low*	Medium	High
Q 2. Have the staff been informed about the transition?	0%	0%	100%	0%	0%	100%
Q 3. Does the NGO/CBO have a JAT (Joint Appraisal Team) score?	47%	53%	0%	0%	67%[2]	33%
Q 4. Has there been communication about empanelment from SACS?	0%	6%	94%	0%	33%	67%
Q 5. Has there been any change in the reporting format, and are you sending any reports to SACS/District AIDS Control Societies?	0%	0%	100%	0%	0%	100%
Q 6. Has there been any change in the TI team structure, and are you following the SACS/NACO guidelines?	0%	0%	100%	0%	17%	83%
Q 9. Is the NGO/CBO following the STI syndromic management guideline of NACO?	0%	29%	71%	0%	0%	100%
Q 10. Does the NGO/CBO procure STI syndromic management medicines as per NACO/SACS guidelines?	24%[3]	18%	59%	50%[3]	33%	17%
Q 11. Has there been any change in the condom procurement process?	12%	18%	71%	67%[4]	0%	33%

Q 12. Has there been any change in the budget as per NACO/SACS guidelines?	0%	0%	100%	0%	0%	100%

* See Section on Explanations for all cutoffs for Low/Medium/High; High means highly aligned

[2] Some TIs completed JAT but had not been informed about the score.

[3] Some TIs have a stock of medicines and therefore had not yet had to procure through NACO.

[4] The condom procurement process did not change for some TIs, while others switched to KSAPS.

	KR 2012			KR 2011		
	Low	Medium	High	Low	Medium	High
Q 7. What is the present ratio of peer educators to HRG?*	0%	24%	76%	0%	0%	100%
Q 8. What is the present ratio of outreach worker to HRG?**	0%	0%	100%	0%	0%	100%

*NACO norm: Ratio of peer educators to HRG is 1: 60

**NACO norm: Ratio of outreach worker to HRG is 1: 250

Q 13 & 14. Have the following staffs received training for the transition OR meetings on the transition as recommended by SACS?	KR 2012 Trainings			KR 2012 Meetings			KR 2011 Trainings & Meetings		
Training for Transition, by Topic* **	None	1 staff	>1 staff	None	1 staff	>1 staff	None	1 staff	>1 staff
TI guidelines	0%	0%	100%	24%	0%	76%	0%	64%	36%
Program management	6%	18%	76%	29%	6%	65%	0%	45%	55%
Outreach planning	18%	0%	82%	47%	0%	53%	9%	9%	82%
Condom programing	18%	0%	82%	47%	0%	53%	36%	27%	36%
Community mobilization	24%	6%	71%	53%	0%	47%	27%	0%	73%
Communication	24%	0%	76%	53%	0%	47%	18%	18%	64%
Counselling	12%	18%	71%	47%	6%	47%	9%	36%	55%
STI management	6%	0%	94%	47%	0%	53%	0%	55%	45%

* Percentages indicate the proportion of TIs in the state where 0, 1 or more than 1 type of staff attended the training for transition.

** Percentages show training that has been received. It does not count training that has been planned but has not yet taken place.

Q 13 & 14. Have the following staffs received training for the transition OR meetings on the transition as recommended by SACS?	KR 2012 Trainings			KR 2012 Meetings			KR 2011 Trainings & Meetings		
Training for Transition, by Staff*	Lowest	Average	Highest	Lowest	Average	Highest	Lowest	Average	Highest
PC/PD	2	6	8	0	4	8	2	5	8
Field Staff	1	5	7	0	4	8	0	4	8
Counsellor/ANM	3	6	8	0	4	8	0	2	5
Doctor	0	1	3	0	1	7	0	1	2
MIS Officer	0	5	7	0	3	8	0	2	8
ORW	2	5	8	0	4	8	0	0	0

* This indicates the lowest, average and highest numbers of types of trainings attended by each staff by state. Greater numbers indicate that staff in the respective category attended multiple types of trainings for the transition

KR 2012

Q 15. Does the NGO/CBO have a buffer in any of the following areas?	No	Yes
Medicines	82%	18%
Condoms	76%	24%
Funds	100%	0%

	KR 2012			
	No flexibility		Flexibility provided	
Q 16. Has there been any flexibility provided from NACO/SACS in the following areas?	Not important	Important	Not important	Important
Reporting	29%	41%	6%	24%
Staffing	12%	41%	6%	41%
Budgets	29%	65%	0%	6%
STI services	12%	35%	12%	41%
Condom programming	29%	24%	6%	41%
Linkages to ICTC	18%	24%	0%	59%

Section 2: TI Alignment - From Interviews

	KR 2012			KR 2011		
	Low	Medium	High	Low	Medium	High
Q 1. Has there been any change in the Avahan method of micro-planning	41%	18%	41%	17%	0%	83%
Q 2. Do all identified hotspots/halting places have condom outlets (TO, NTO, CVM)?	35%	59%	6%	0%	67%	33%

	KR 2012		KR 2011	
Q 3. Does the NGO/CBO have a linkage with the following government services?	Most cases are referred	Most cases referred receive these services*	Most cases are referred	Most cases referred receive these services*
Government ICTC	100%	100%	100%	100%
ART centres	100%	100%	100%	100%
TB screening centres	100%	100%	100%	0%

* Percentages show NGO/CBOs where most cases are referred to government services AND those referred receive these services

	KR 2012			KR 2011		
Q 4. Which places do people go for STI services?	1st Choice (most preferred)	2nd Choice	3rd Choice	1st Choice (most preferred)	2nd Choice	3rd Choice
On-site clinic, Part time	0%	6%	18%	67%	0%	0%
On-site clinic, Full time	41%	12%	29%	0%	0%	17%
Government hospital clinic	24%	53%	24%	33%	50%	17%
Preferred private practitioner	35%	29%	29%	0%	50%	33%
None	0%	0%	0%	0%	0%	33%

	KR 2012			KR 2011		
	Low	Medium	High	Low	Medium	High
Q 5. Has the NGO/CBO been able to form groups at the community level?	0%	0%	100%	0%	0%	100%
Q 6. Have community members at the hotspots formed crisis response committees?	0%	12%	88%	0%	33%	67%

Section 3: TI Alignment - From CMIS Avahan*

	KR 2012						
	Low	Medium	High	Don't know / NA	Data not available	# TIs with data	Average value
Q 1. What is the coverage of identified HRGs with regular contact (two contacts each month)?	0%	0%	100%	0%	0 (0%)	17	97%
Q 2. What is the coverage of free condom supply among the identified HRG by NGO/CBO?	0%	12%	6%	82%	17 (100%)	0	-
Q 3. What percentage of HRGs who are referred actually visit the ICTC?	35%	18%	47%	0%	2 (12%)	15	78%
Q 4. What is the coverage of syndromic management for HRGs with STI?	41%	18%	41%	0%	5 (29%)	12	99%

	KR 2011						
	Low	Medium	High	Don't know	Data not available	# TIs with data	Average value
Q 1. What is the coverage of identified HRGs with regular contact (two contacts each month)?*	0%	0%	100%	0%	0 (0%)	6	75%
Q 2. What is the coverage of free condom supply among the identified HRG by NGO/CBO?**	0%	67%	33%	0%	0 (0%)	6	97%
Q 3. What percentage of HRGs who are referred actually visit the ICTC?***	17%	50%	33%	0%	0 (0%)	6	103%
Q 4. What is the coverage of syndromic management for HRGs with STI? ****	0%	50%	50%	0%	0 (0%)	6	115%

*NACO norm: 60% of identified HRGs have at least two contacts each month
**NACO norm: 100% of identified HRGs have access to free condoms
***NACO norm: 100% of HRGs who are referred should visit the ICTC
****NACO norm: 100% of HRGs with STI syndromes receive treatment

Transition Readiness Dashboard

From Round 1 & 2 Transition Readiness Assessment 2011 & 2012

Evaluative Assessment of Phase II of the Avahan Program: Transition and Influence Goals

4. Results of HRGs in TAMIL NADU

	TN 2012	TN 2011
Number of TIs interviewed in state	7	4

	TN 2012		TN 2011	
	NGO	CBO	NGO	CBO
Are you a NGO or CBO?	2	5	2	2

Section 1: NGO/CBO Capacity

	TN 2012			TN 2011		
	None	Societies Registration Act	States Trusts Acts	None	Societies Registration Act	States Trusts Acts
Q 1. What is the legal status of the organization? (mention the registration details under the societies / public trusts as applicable)	0%	29%	71%	0%	25%	75%

	TN 2012		TN 2011	
	No	Yes	No	Yes
Q 1.1. Does the organization have a Foreign Contribution (Regulation) Act 1976 account?	43%	57%	75%	25%

	TN 2012			TN 2011		
	Low*	Medium	High	Low*	Medium	High
Q 2. Have the staff been informed about the transition?	0%	71%	29%	0%	50%	50%
Q 3. Does the NGO/CBO have a JAT (Joint Appraisal Team) score?	71%	14%	14%	75%[1]	25%	0%
Q 4. Has there been communication about empanelment from SACS?	71%	29%	0%	0%	25%	75%
Q 5. Has there been any change in the reporting format, and are you sending any reports to SACS/District AIDS Control Societies?	57%	14%	29%	0%	0%	100%
Q 6. Has there been any change in the TI team structure, and are you following the SACS/NACO guidelines?	0%	0%	100%	0%	0%	100%
Q 9. Is the NGO/CBO following the STI syndromic management guideline of NACO?	14%	0%	86%	0%	0%	100%
Q 10. Does the NGO/CBO procure STI syndromic management medicines as per NACO/SACS guidelines?	29%	0%	71%	0%	0%	100%
Q 11. Has there been any change in the condom procurement process?	14%	14%	71%	0%	0%	100%
Q 12. Has there been any change in the budget as per NACO/SACS guidelines?	0%	57%	43%	0%	0%	100%

* See Section on Explanations for all cutoffs for Low/Medium/High; High means highly aligned

[1] JAT had not taken place in some TIs

	TN 2012			TN 2011		
	Low	Medium	High	Low	Medium	High
Q 7. What is the present ratio of peer educators to HRG?*	0%	0%	100%	0%	0%	100%
Q 8. What is the present ratio of outreach worker to HRG?**	0%	0%	100%	0%	0%	100%

*NACO norm: Ratio of peer educators to HRG is 1: 60

**NACO norm: Ratio of outreach worker to HRG is 1: 250

Q 13 & 14. Have the following staffs received training for the transition OR meetings on the transition as recommended by SACS?	TN 2012 Trainings			TN 2012 Meetings			TN 2011 Trainings & Meetings		
Training for Transition, by Topic* **	None	1 staff	>1 staff	None	1 staff	>1 staff	None	1 staff	>1 staff
TI guidelines	14%	0%	86%	0%	0%	100%	0%	64%	36%
Program management	0%	0%	100%	0%	14%	86%	0%	45%	55%
Outreach planning	0%	0%	100%	0%	0%	100%	9%	9%	82%
Condom programing	0%	0%	100%	0%	0%	100%	36%	27%	36%
Community mobilization	0%	0%	100%	0%	14%	86%	27%	0%	73%
Communication	0%	14%	86%	0%	14%	86%	18%	18%	64%
Counselling	0%	0%	100%	0%	29%	71%	9%	36%	55%
STI management	0%	0%	100%	0%	0%	100%	0%	55%	45%

* Percentages indicate the proportion of TIs in the state where 0, 1 or more than 1 type of staff attended the training for transition.

** Percentages show training that has been received. It does not count training that has been planned but has not yet taken place.

Q 13 & 14. Have the following staffs received training for the transition OR meetings on the transition as recommended by SACS?	TN 2012 Trainings			TN 2012 Meetings			TN 2011 Trainings & Meetings		
Training for Transition, by Staff*	Lowest	Average	Highest	Lowest	Average	Highest	Lowest	Average	Highest
PC/PD	6	8	8	5	8	8	2	5	8
Field Staff	3	6	8	3	6	8	0	4	8
Counsellor/ANM	2	5	8	2	5	8	0	2	5
Doctor	0	3	5	0	3	5	0	1	2
MIS Officer	2	6	8	2	5	8	0	2	8
ORW	4	6	12	4	6	12	0	0	0

* This indicates the lowest, average and highest numbers of types of trainings attended by each staff by state. Greater numbers indicate that staff in the respective category attended multiple types of trainings for the transition

	TN 2012	
Q 15. Does the NGO/CBO have a buffer in any of the following areas?	No	Yes
Medicines	0%	100%
Condoms	0%	100%
Funds	43%	57%

Q 16. Has there been any flexibility provided from NACO/SACS in the following areas?	TN 2012			
	No flexibility		Flexibility provided	
	Not important	Important	Not important	Important
Reporting	57%	29%	0%	14%
Staffing	29%	29%	14%	29%
Budgets	14%	29%	0%	57%
STI services	29%	29%	0%	43%
Condom programming	43%	14%	0%	43%
Linkages to ICTC	0%	14%	0%	86%

Section 2: TI Alignment - From Interviews

	TN 2012			TN 2011		
	Low	Medium	High	Low	Medium	High
Q 1. Has there been any change in the Avahan method of micro-planning	0%	29%	71%	50%[2]	0%	50%
Q 2. Do all identified hotspots/halting places have condom outlets (TO, NTO, CVM)?	29%	43%	29%	0%	0%	100%

[2] Avahan method of microplanning continued to be implemented

Q 3. Does the NGO/CBO have a linkage with the following government services?	TN 2012		TN 2011	
	Most cases are referred	Most cases referred receive these services*	Most cases are referred	Most cases referred receive these services*
Government ICTC	58%	29%[3]	100%	25%[3]
ART centres	100%	29%[3]	100%	0%[3]
TB screening centres	72%	29%[3]	100%	25%[3]

* Percentages show NGO/CBOs where most cases are referred to government services AND those referred receive these services

[3] PC/PDs in some TIs did not perceive that most people who are referred actually receive the services.

Q 4. Which places do people go for STI services?	TN 2012			TN 2011		
	1st Choice (most preferred)	2nd Choice	3rd Choice	1st Choice (most preferred)	2nd Choice	3rd Choice
On-site clinic, Part time	43%	0%	0%	0%	0%	0%
On-site clinic, Full time	57%	0%	0%	100%	0%	0%
Government hospital clinic	0%	57%	43%	0%	100%	0%
Preferred private practitioner	0%	43%	57%	0%	0%	75%
None	0%	0%	0%	0%	0%	25%

	TN 2012			TN 2011		
	Low	Medium	High	Low	Medium	High
Q 5. Has the NGO/CBO been able to form groups at the community level?	14%	0%	86%	0%	0%	100%

Q 6. Have community members at the hotspots formed crisis response committees?	0%	43%	57%	0%	50%	50%

Section 3: TI Alignment - From CMIS Avahan

	TN 2012						
	Low	Medium	High	Don't know / NA	Data not available	# TIs with data	Average value
Q 1. What is the coverage of identified HRGs with regular contact (two contacts each month)?	0%	14%	86%	0%	0 (0%)	7	78%
Q 2. What is the coverage of free condom supply among the identified HRG by NGO/CBO?	0%	29%	71%	0%	6 (86%)	1	-
Q 3. What percentage of HRGs who are referred actually visit the ICTC?	14%	43%	43%	0%	0 (0%)	7	82%
Q 4. What is the coverage of syndromic management for HRGs with STI?	29%	0%	71%	0%	2 (29%)	5	100%

	TN 2011						
	Low	Medium	High	Don't know	Data not available	# TIs with data	Average value
Q 1. What is the coverage of identified HRGs with regular contact (two contacts each month)?*	25%	25%	50%	0%	0 (0%)	4	69%
Q 2. What is the coverage of free condom supply among the identified HRG by NGO/CBO?**	0%	0%	100%	0%	0 (0%)	4	112%
Q 3. What percentage of HRGs who are referred actually visit the ICTC?***	0%	50%	50%	0%	0 (0%)	4	90%
Q 4. What is the coverage of syndromic management for HRGs with STI? ****	0%	0%	100%	0%	2 (50%)	2	100%

*NACO norm: 60% of identified HRGs have at least two contacts each month
**NACO norm: 100% of identified HRGs have access to free condoms
***NACO norm: 100% of HRGs who are referred should visit the ICTC
****NACO norm: 100% of HRGs with STI syndromes receive treatment

Transition Readiness Dashboard

From Round 1 & 2 Transition Readiness Assessment 2011 & 2012

Evaluative Assessment of Phase II of the Avahan Program: Transition and Influence Goals

5. Results of HRGs in MAHARASHTRA

	MH 2012	MH 2011
Number of TIs interviewed in state	13	6

	MH 2012		MH 2011	
	NGO	CBO	NGO	CBO
Are you a NGO or CBO?	11	2	6	0

Section 1: NGO/CBO Capacity

	MH 2012			MH 2011		
	None	Societies Registration Act	States Trusts Acts	None	Societies Registration Act	States Trusts Acts
Q 1. What is the legal status of the organization? (mention the registration details under the societies / public trusts as applicable)	0%	62%	38%	0%	83%	17%

	MH 2012		MH 2011	
	No	Yes	No	Yes
Q 1.1. Does the organization have a Foreign Contribution (Regulation) Act 1976 account?	23%	77%	17%	83%

	MH 2012			MH 2011		
	Low*	Medium	High	Low*	Medium	High
Q 2. Have the staff been informed about the transition?	0%	46%	54%	0%	83%	17%
Q 3. Does the NGO/CBO have a JAT (Joint Appraisal Team) score?	31%	23%	46%	33%[1]	67%	0%
Q 4. Has there been communication about empanelment from SACS?	23%	23%	54%	0%	67%	33%
Q 5. Has there been any change in the reporting format, and are you sending any reports to SACS/District AIDS Control Societies?	0%	0%	100%	0%	17%	83%
Q 6. Has there been any change in the TI team structure, and are you following the SACS/NACO guidelines?	0%	15%	85%	0%	17%	83%
Q 9. Is the NGO/CBO following the STI syndromic management guideline of NACO?	0%	15%	85%	33%[2]	0%	67%
Q 10. Does the NGO/CBO procure STI syndromic management medicines as per NACO/SACS guidelines?	46%	15%	38%	67%[3]	0%	33%
Q 11. Has there been any change in the condom procurement process?	38%	15%	46%	50%[4]	33%	17%

Q 12. Has there been any change in the budget as per NACO/SACS guidelines?	8%	15%	77%	0%	17%	83%

* See Section on Explanations for all cutoffs for Low/Medium/High; High means highly aligned

[1] Some TIs did not have JAT, while others completed JAT but had not been informed about the score.

[2] Some TIs still followed Avahan guidelines.

[3] Some TIs had a buffer stock at hand and planned to use the Avahan supply chain for some time.

[4] There were no changes in the condom procurement process for some TIs.

	MH 2012			MH 2011		
	Low	Medium	High	Low	Medium	High
Q 7. What is the present ratio of peer educators to HRG?*	0%	0%	100%	0%	17%	83%
Q 8. What is the present ratio of outreach worker to HRG?**	0%	31%	69%	17%	33%	50%

*NACO norm: Ratio of peer educators to HRG is 1: 60

**NACO norm: Ratio of outreach worker to HRG is 1: 250

Q 13 & 14. Have the following staffs received training for the transition OR meetings on the transition as recommended by SACS?	MH 2012 Trainings			MH 2012 Meetings			MH 2011 Trainings & Meetings		
Training for Transition, by Topic* **	None	1 staff	>1 staff	None	1 staff	>1 staff	None	1 staff	>1 staff
TI guidelines	0%	0%	100%	8%	0%	92%	0%	64%	36%
Program management	0%	0%	100%	8%	0%	92%	0%	45%	55%
Outreach planning	0%	0%	100%	8%	0%	92%	9%	9%	82%
Condom programing	0%	0%	100%	8%	0%	92%	36%	27%	36%
Community mobilization	0%	0%	100%	8%	0%	92%	27%	0%	73%
Communication	0%	0%	100%	8%	0%	92%	18%	18%	64%
Counselling	0%	0%	100%	8%	0%	92%	9%	36%	55%
STI management	0%	0%	100%	8%	0%	92%	0%	55%	45%

* Percentages indicate the proportion of TIs in the state where 0, 1 or more than 1 type of staff attended the training for transition.

** Percentages show training that has been received. It does not count training that has been planned but has not yet taken place.

Q 13 & 14. Have the following staffs received training for the transition OR meetings on the transition as recommended by SACS?	MH 2012 Trainings			MH 2012 Meetings			MH 2011 Trainings & Meetings		
Training for Transition, by Staff*	Lowest	Average	Highest	Lowest	Average	Highest	Lowest	Average	Highest
PC/PD	4	6	12	0	7	8	2	5	8
Field Staff	4	6	12	0	6	8	0	4	8
Counsellor/ANM	4	7	12	0	7	8	0	2	5
Doctor	4	6	12	0	6	8	0	1	2
MIS Officer	4	6	12	0	6	8	0	2	8
ORW	4	6	12	0	6	8	0	0	0

* This indicates the lowest, average and highest numbers of types of trainings attended by each staff by state. Greater numbers indicate that staff in the respective category attended multiple types of trainings for the transition

	MH 2012	
Q 15. Does the NGO/CBO have a buffer in any of the following areas?	No	Yes
Medicines	38%	62%
Condoms	38%	62%
Funds	31%	69%

	MH 2012			
	No flexibility		Flexibility provided	
Q 16. Has there been any flexibility provided from NACO/SACS in the following areas?	Not important	Important	Not important	Important
Reporting	0%	92%	0%	8%
Staffing	0%	92%	0%	8%
Budgets	0%	85%	0%	15%
STI services	8%	62%	0%	31%
Condom programming	8%	85%	0%	8%
Linkages to ICTC	8%	69%	0%	23%

Section 2: TI Alignment - From Interviews

	MH 2012			MH 2011		
	Low	Medium	High	Low	Medium	High
Q 1. Has there been any change in the Avahan method of micro-planning	31%[5]	15%	54%	33%[5]	0%	67%
Q 2. Do all identified hotspots/halting places have condom outlets (TO, NTO, CVM)?	23%	54%	23%	0%	17%	83%

[5] Avahan method of microplanning continued to be implemented

	MH 2012		MH 2011	
Q 3. Does the NGO/CBO have a linkage with the following government services?	Most cases are referred	Most cases referred receive these services*	Most cases are referred	Most cases referred receive these services*
Government ICTC	100%	69%	83%	33%
ART centres	100%	62%	100%	33%
TB screening centres	100%	62%	100%	50%

* Percentages show NGO/CBOs where most cases are referred to government services AND those referred receive these services

	MH 2012			MH 2011		
Q 4. Which places do people go for STI services?	1st Choice (most preferred)	2nd Choice	3rd Choice	1st Choice (most preferred)	2nd Choice	3rd Choice
On-site clinic, Part time	31%	8%	8%	50%	17%	0%
On-site clinic, Full time	15%	8%	46%	33%	33%	0%
Government hospital clinic	0%	77%	8%	17%	33%	17%
Preferred private practitioner	54%	8%	38%	0%	17%	83%

None	0%	0%	0%	0%	0%	0%

	MH 2012			MH 2011		
	Low	Medium	High	Low	Medium	High
Q 5. Has the NGO/CBO been able to form groups at the community level?	0%	31%	69%	0%	0%	100%
Q 6. Have community members at the hotspots formed crisis response committees?	15%	15%	69%	0%	0%	100%

Section 3: TI Alignment - From CMIS Avahan

	MH 2012						
	Low	Medium	High	Don't know / NA	Data not available	# TIs with data	Average value
Q 1. What is the coverage of identified HRGs with regular contact (two contacts each month)?	0%	8%	92%	0%	0 (0%)	13	95%
Q 2. What is the coverage of free condom supply among the identified HRG by NGO/CBO?	-	-	-	-	13 (100%)	0	-
Q 3. What percentage of HRGs who are referred actually visit the ICTC?	23%	15%	62%	0%	3 (23%)	10	95%
Q 4. What is the coverage of syndromic management for HRGs with STI?	15%	23%	62%	0%	2 (15%)	11	108%

	MH 2011						
	Low	Medium	High	Don't know	Data not available	# TIs with data	Average value
Q 1. What is the coverage of identified HRGs with regular contact (two contacts each month)?*	0%	0%	100%	0%	0 (0%)	6	113%
Q 2. What is the coverage of free condom supply among the identified HRG by NGO/CBO?**	-	-	-	-	6 (100%)	0	-
Q 3. What percentage of HRGs who are referred actually visit the ICTC?***	0%	0%	100%	0%	1 (17%)	5	100%
Q 4. What is the coverage of syndromic management for HRGs with STI? ****	0%	40%	60%	0%	1 (17%)	5	130%

*NACO norm: 60% of identified HRGs have at least two contacts each month
**NACO norm: 100% of identified HRGs have access to free condoms
***NACO norm: 100% of HRGs who are referred should visit the ICTC
****NACO norm: 100% of HRGs with STI syndromes receive treatment

Transition Readiness Dashboard

From Round 1 & 2 Transition Readiness Assessment 2011 & 2012

Evaluative Assessment of Phase II of the Avahan Program: Transition and Influence Goals

6. Buffer & Flexibility by State

Q 15. Does the NGO/CBO have a buffer in any of the following areas?	Buffer Provided[A]			
	AP	KR	TN	MH
Medicines[B]	94%	18%	100%	62%
Condoms[C]	13%	24%	100%	62%
Funds[D]	0%	0%	57%	69%

[A] Some TIs reported not having a buffer in all areas (especially in KR). Advanced planning often helped in these TIs where items were received 3 months in advance. 1 TI noted shortage of medicines without a buffer. Another TI noted taking from a trust when funds run out.

[B] TIs reported having a medicine buffer ranging from 2% to 1, 3, and 6 months. The majority (17 TIs) reported having a medicine buffer of 6 months.

[C] Some TIs reported having a condom buffer ranging from 5% or 60,000 condoms to 1, 3, and 6 months. 3 TIs reported having a buffer of 6 months.

[D] Few TIs reported having buffer for funds. Release of funds ranged by TI, from yearly, every 6 months, quarterly, to every 3 months.

Q 16. Has there been any flexibility provided from NACO/SACS in the following areas?	No flexibility & Not important					No flexibility, but Important[A]				
	AP	KR	TN	MH	Average	AP	KR	TN	MH	Average
Reporting	69%	29%	57%	0%	39%	31%	41%	29%	92%	48%
Staffing	31%	12%	29%	0%	18%	69%	41%	29%	92%	58%
Budgets	6%	29%	14%	0%	12%	94%	65%	29%	85%	68%
STI services	88%	12%	29%	8%	34%	13%	35%	29%	62%	35%
Condom programming	94%	29%	43%	8%	44%	6%	24%	14%	85%	32%
Linkages to ICTC	75%	18%	0%	8%	25%	25%	24%	14%	69%	33%

[A] Some TIs reported being compelled to follow all norms strictly. Some noted that SLPs used to help a lot; now KPs have to manage on their own.

Q 16. Has there been any flexibility provided from NACO/SACS in the following areas?	Flexibility provided, but Not important					Flexibility provided & Important[B]				
	AP	KR	TN	MH	Average	AP	KR	TN	MH	Average
Reporting	0%	6%	0%	0%	2%	0%	24%	14%	8%	12%

Staffing	0%	6%	14%	0%	5%	0%	41%	29%	8%	20%
Budgets	0%	0%	0%	0%	0%	0%	6%	57%	15%	20%
STI services	0%	12%	0%	0%	3%	0%	41%	43%	31%	29%
Condom programming	0%	6%	0%	0%	2%	0%	41%	43%	8%	23%
Linkages to ICTC	0%	0%	0%	0%	0%	0%	59%	86%	23%	42%

[B] ***Some TIs (especially in TN) reported asking SACS for flexibility about providing more counsellors, where flexibility was granted.***

Q 16. Has there been any flexibility provided from NACO/SACS in the following areas?	**Flexibility Provided**					**Flexibility Important**[C]				
	AP	KR	TN	MH	Average	AP	KR	TN	MH	Average
Reporting	0%	8%	30%	14%	13%	31%	100%	65%	43%	60%
Staffing	0%	8%	47%	43%	25%	69%	100%	82%	58%	77%
Budgets	0%	15%	6%	57%	20%	94%	100%	71%	86%	88%
STI services	0%	31%	53%	43%	32%	13%	93%	76%	72%	64%
Condom programming	0%	8%	47%	43%	25%	6%	93%	65%	57%	55%
Linkages to ICTC	0%	23%	59%	86%	42%	25%	92%	83%	100%	75%

[C] ***Many TIs reported flexibility to be important especially for staffing and budgets.***

Transition Readiness Dashboard

From Round 1 & 2 Transition Readiness Assessment 2011 & 2012

Evaluative Assessment of Phase II of the Avahan Program: Transition and Influence Goals

7. Results of HRGs by NGO/CBO

	2012		2011	
	NGO	CBO	NGO	CBO
Are you a NGO or CBO?	34	19	17	10

	2012							
	NGO				CBO			
	AP	KR	TN	MH	AP	KR	TN	MH
In which state are you located?	14	7	2	11	2	10	5	2

	2011					
	NGO			CBO		
	AP*	TN	MH	AP	KR	TN
In which state are you located?	9	2	6	2	6	2

*AP: Andra Pradesh, KR: Karnataka, TN: Tamil Nadu, MH: Maharashtra

Section 1: NGO/CBO Capacity

	2012						2011					
	NGO			CBO			NGO			CBO		
	None	Societies Registration Act	States Trusts Acts	None	Societies Registration Act	States Trusts Acts	None	Societies Registratio n Act	States Trusts Acts	None	Societies Registration Act	States Trusts Acts
Q 1. What is the legal status of the organization? (mention the registration details under the societies / public trusts as applicable)	0%	88%*	12%	0%	68%**	32%	0%	88%	12%	0%	80%	20%

Among 19 NGOs of 2012 transition that mentioned the year of registration, 5 were registered in the 1980s, 5 in the 1990s and 9 between 2000-2008.

*****Among 8 CBOs of 2012 transition that mentioned the year of registration, 2 were registered in the 1980s, 4 in the 1990s and 2 between 2000-2010.***

	2012				2011			
	NGO		CBO		NGO		CBO	
	No	Yes	No	Yes	No	Yes	No	Yes
Q 1.1. Does the organization have a Foreign Contribution (Regulation) Act 1976 account?	3%	97%*	53%	47%**	12%	88%	80%	20%

Among 8 NGOs of 2012 transition that mentioned having FCRA, 2 were registered in the 1980s, 1 in the 1990s and 5 between 2000-2006.

Among 5 CBOs of 2012 transition that mentioned having FCRA, all 5 were registered between 2007-2011.

	2012						2011					
	NGO			CBO			NGO			CBO		
	Low*	Medium	High	Low	Medium	High	Low*	Medium	High	Low	Medium	High
Q 2. Have the staff been informed about the transition?	0%	32%	68%	0%	26%	74%	0%	35%	65%	0%	10%	90%
Q 3. Does the NGO/CBO have a JAT (Joint Appraisal Team) score?	15%	47%	38%	63%	26%	11%	18%	35%	47%	20%	40%	40%
Q 4. Has there been communication about empanelment from SACS?	6%	15%	79%	31%	16%	53%	0%	24%	76%	0%	30%	70%
Q 5. Has there been any change in the reporting format, and are you sending any reports to SACS/District AIDS Control Societies?	3%	0%	97%	16%	5%	79%	0%	6%	94%	0%	0%	100%
Q 6. Has there been any change in the TI team structure, and are you following the SACS/NACO guidelines?	0%	6%	94%	0%	0%	100%	0%	6%	94%	0%	10%	90%
Q 9. Is the NGO/CBO following the STI syndromic management guideline of NACO?	0%	0%	100%	5%	37%	58%	24%	0%	76%	0%	0%	100%
Q 10. Does the NGO/CBO procure STI syndromic management medicines as per NACO/SACS guidelines?	65%**	3%	32%	32%**	21%	47%	41%**	0%	59%	30%**	20%	50%
Q 11. Has there been any change in the condom procurement process?	15%	6%	79%	16%	21%	63%	18%	12%	71%	40%	0%	60%
Q 12. Has there been any change in the budget as per NACO/SACS guidelines?	3%	3%	94%	0%	26%	74%	0%	12%	88%	0%	0%	100%

* See Section on Explanations for all cutoffs for Low/Medium/High; High means highly aligned

** Many of these TIs indicated that Avahan supply chain is still in place for medicines because they have a buffer stock in hand

	2012						2011					
	NGO			CBO			NGO			CBO		
	Low*	Medium	High	Low	Medium	High	Low*	Medium	High	Low	Medium	High
Q 7. What is the present ratio of peer educators to HRG?*	0%	0%	100%	0%	21%	79%	0%	6%	94%	0%	0%	100%

Q 8. What is the present ratio of outreach worker to HRG?**	0%	12%	88%	0%	5%	95%	6%	12%	82%	0%	0%	100%

*NACO norm: Ratio of peer educators to HRG is 1: 60

**NACO norm: Ratio of outreach worker to HRG is 1: 250

	2012 Trainings						2012 Meetings						2011					
Q 13 & 14. Have the following staffs received training for the transition OR meetings on the transition as recommended by SACS?	NGO			CBO			NGO			CBO			NGO			CBO		
	None	1 staff	>1 staff	None	1 staff	>1 staff	None	1 staff	>1 staff	None	1 staff	>1 staff	None	1 staff	>1 staff	None	1 staff	>1 staff
TI guidelines	9%	24%	68%	11%	5%	84%	44%	3%	53%	16%	5%	79%	18%	41%	41%	40%	10%	50%
Program management	12%	24%	65%	5%	16%	79%	44%	6%	50%	21%	11%	68%	12%	0%	88%	70%	10%	20%
Outreach planning	21%	21%	59%	5%	5%	89%	44%	3%	53%	37%	0%	63%	24%	0%	76%	60%	30%	10%
Condom programing	26%	15%	59%	5%	5%	89%	47%	3%	50%	37%	0%	63%	35%	18%	47%	60%	30%	10%
Community mobilization	38%	3%	59%	5%	11%	84%	53%	3%	44%	37%	0%	63%	29%	6%	65%	60%	10%	30%
Communication	29%	15%	56%	11%	11%	79%	53%	0%	47%	37%	5%	58%	18%	18%	65%	70%	0%	30%
Counselling	41%	9%	50%	11%	5%	84%	50%	3%	47%	37%	11%	53%	29%	35%	35%	70%	0%	30%
STI management	26%	6%	68%	11%	0%	89%	47%	3%	50%	37%	0%	63%	18%	0%	82%	70%	10%	20%

* Percentages indicate the proportion of TIs in the state where 0, 1 or more than 1 type of staff attended the training for transition.

** Percentages show training that has been received. It does not count training that has been planned but has not yet taken place.

	2012 Trainings						2012 Meetings						2011					
Q 13 & 14. Have the following staffs received training for the transition OR meetings on the transition as recommended by SACS?	NGO			CBO			NGO			CBO			NGO			CBO		
	Lowest	Average	Highest	Lowest	Average	Highest	Lowest	Average	Highest	Lowest	Average	Highest	Lowest	Average	Highest	Lowest	Average	Highest
PC/PD	0	5	12	0	6	8	0	4	8	0	5	8	0	4	7	0	2	8
Field Staff	0	3	12	0	5	8	0	3	8	0	4	8	0	2	7	0	1	8
Counsellor/ANM	0	4	12	0	5	8	0	3	8	0	4	8	0	2	7	0	1	5
Doctor	0	3	12	0	2	5	0	2	8	0	2	7	0	2	6	0	0	1
MIS Officer	0	4	12	0	4	8	0	3	8	0	4	8	0	2	7	0	2	8
ORW	0	4	12	0	5	12	0	3	8	0	5	12	0	1	7	0	0	1

* This indicates the lowest, average and highest numbers of types of trainings attended by each staff by state. Greater numbers indicate that staff in the respective category attended multiple types of trainings for the transition

	2012			
	NGO		CBO	
Q 15. Does the NGO/CBO have a buffer in any of the following areas?	No	Yes	No	Yes
Medicines	26%	74%	58%	42%
Condoms	62%	38%	58%	42%
Funds	68%	32%	89%	11%

	2012							
	NGO				CBO			
	No flexibility		Flexibility provided		No flexibility		Flexibility provided	
Q 16. Has there been any flexibility provided from NACO/SACS in the following areas?	Not important	Important	Not important	Important	Not important	Important	Not important	Important
Reporting	44%	44%	0%	12%	26%	58%	5%	11%
Staffing	21%	68%	3%	9%	11%	47%	5%	37%
Budgets	9%	76%	0%	15%	21%	68%	0%	11%
STI services	44%	35%	3%	18%	21%	32%	5%	42%
Condom programming	56%	35%	3%	9%	32%	26%	0%	42%
Linkages to ICTC	38%	35%	0%	26%	16%	32%	0%	53%

Section 2: TI Alignment - From Interviews

	2012						2011					
	NGO			CBO			NGO			CBO		
	Low	Medium	High	Low	Medium	High	Low	Medium	High	Low	Medium	High
Q 1. Has there been any change in the Avahan method of micro-planning	15%	20%	65%	32%	10%	58%	18%	0%	82%	20%	0%	80%
Q 2. Do all identified hotspots/halting places have condom outlets (TO, NTO, CVM)?	18%	70%	12%	26%	63%	11%	0%	6%	94%	0%	40%	60%

	2012				2011			
	NGO		CBO		NGO		CBO	
Q 3. Does the NGO/CBO have a linkage with the following government services?	Most cases are referred	Most cases referred receive these services*	Most cases are referred	Most cases referred receive these services*	Most cases are referred	Most cases referred receive these services*	Most cases are referred	Most cases referred receive these services*
Government ICTC	100%	85%	84%	79%	94%	18%	100%	60%
ART centres	100%	82%	100%	79%	100%	12%	100%	60%

TB screening centres	100%	82%	89%	79%	100%	24%	100%	60%

* Percentages show NGO/CBOs where most cases are referred to government services AND those referred receive these services

	2012						2011					
	NGO			CBO			NGO			CBO		
Q 4. Which places do people go for STI services?	1st Choice (most preferred)	2nd Choice	3rd Choice	1st Choice (most preferred)	2nd Choice	3rd Choice	1st Choice (most preferred)	2nd Choice	3rd Choice	1st Choice (most preferred)	2nd Choice	3rd Choice
On-site clinic, Part time	27%	3%	9%	11%	5%	5%	24%	6%	0%	40%	0%	0%
On-site clinic, Full time	41%	6%	26%	58%	5%	21%	65%	12%	0%	40%	0%	10%
Government hospital clinic	6%	79%	9%	10%	58%	32%	6%	53%	29%	20%	60%	20%
Preferred private practitioner	26%	12%	56%	21%	32%	42%	6%	30%	65%	0%	40%	40%
None	0%	0%	0%	0%	0%	0%	0%	0%	6%	0%	0%	30%

	2012						2011					
	NGO			CBO			NGO			CBO		
	Low	Medium	High	Low	Medium	High	Low	Medium	High	Low	Medium	High
Q 5. Has the NGO/CBO been able to form groups at the community level?	3%	9%	88%	0%	11%	89%	0%	0%	100%	0%	0%	100%
Q 6. Have community members at the hotspots formed crisis response committees?	0%	15%	85%	10%	11%	79%	0%	12%	88%	0%	20%	80%

Section 3: TI Alignment - From CMIS Avahan

	2012									
	NGO					CBO				
	Low	Medium	High	Don't know / NA	Data not available	Low	Medium	High	Don't know	Data not available
Q 1. What is the coverage of identified HRGs with regular contact (two contacts each month)?	0%	3%	97%	0%	0 (0%)	0%	11%	89%	0%	0 (0%)
Q 2. What is the coverage of free condom supply among the identified HRG by NGO/CBO?	0%	18%	29%	53%	6 (35%)	5%	32%	63%	0%	1 (10%)
Q 3. What percentage of HRGs who are referred actually visit the ICTC?	56%	18%	26%	0%	5 (29%)	21%	16%	63%	0%	1 (10%)
Q 4. What is the coverage of syndromic management for HRGs with STI?	18%	24%	58%	0%	4 (24%)	32%	5%	63%	0%	1 (10%)

	2011									
	NGO					CBO				
	Low	Medium	High	Don't know	Data not available	Low	Medium	High	Don't know	Data not available
Q 1. What is the coverage of identified HRGs with regular contact (two contacts each month)?*	0%	6%	94%	0%	0 (0%)	11%	0%	89%	0%	1 (10%)
Q 2. What is the coverage of free condom supply among the identified HRG by NGO/CBO?**	0%	18%	82%	0%	6 (35%)	0%	44%	56%	0%	1 (10%)
Q 3. What percentage of HRGs who are referred actually visit the ICTC?***	8%	25%	67%	0%	5 (29%)	11%	44%	44%	0%	1 (10%)
Q 4. What is the coverage of syndromic management for HRGs with STI? ****	8%	15%	77%	0%	4 (24%)	0%	33%	66%	0%	1 (10%)

*NACO norm: 60% of identified HRGs have at least two contacts each month
**NACO norm: 100% of identified HRGs have access to free condoms
***NACO norm: 100% of HRGs who are referred should visit the ICTC
****NACO norm: 100% of HRGs with STI syndromes receive treatment

Transition Readiness Dashboard

From Round 1 & 2 Transition Readiness Assessment 2011 & 2012

Evaluative Assessment of Phase II of the Avahan Program: Transition and Influence Goals

8. Results by HRG Category

	2012			2011		
	FSW	MSM	Both	FSW	MSM	Both
For whom do you provide services?	20	11	22	14	2	11

	2012					
	FSW		MSM		Both	
	NGO	CBO	NGO	CBO	NGO	CBO
Are you a NGO or CBO?	13	7	5	6	16	6

	2012											
	FSW				MSM				Both			
	AP	KR	TN	MH	AP	KR	TN	MH	AP	KR	TN	MH
In which state are you located?	1	10	2	7	0	6	0	5	15	1	5	1

*AP: Andra Pradesh, KR: Karnataka, TN: Tamil Nadu, MH: Maharashtra

Section 1: NGO/CBO Capacity

	2012								
	FSW			MSM			Both		
	None	Societies Registration Act	States Trusts Acts	None	Societies Registration Act	States Trusts Acts	None	Societies Registratio n Act	States Trusts Acts
Q 1. What is the legal status of the organization? (mention the registration details under the societies / public trusts as applicable)	0%	80%	20%	0%	73%	27%	0%	86%	14%

	2012					
	FSW		MSM		Both	
	No	Yes	No	Yes	No	Yes
Q 1.1. Does the organization have a Foreign Contribution (Regulation) Act 1976 account?	15%	85%	27%	73%	23%	77%

	2012								
	FSW			MSM			Both		
	Low*	Medium	High	Low	Medium	High	Low*	Medium	High
Q 2. Have the staff been informed about the transition?	0%	35%	65%	0%	9%	91%	0%	36%	64%
Q 3. Does the NGO/CBO have a JAT (Joint Appraisal Team) score?	50%	25%	25%	36%	45%	18%	14%	50%	36%
Q 4. Has there been communication about empanelment from SACS?	10%	10%	80%	18%	27%	55%	18%	14%	68%
Q 5. Has there been any change in the reporting format, and are you sending any reports to SACS/District AIDS Control Societies?	10%	0%	90%	0%	0%	100%	9%	5%	86%
Q 6. Has there been any change in the TI team structure, and are you following the SACS/NACO guidelines?	0%	10%	90%	0%	0%	100%	0%	0%	100%
Q 9. Is the NGO/CBO following the STI syndromic management guideline of NACO?	0%	10%	90%	0%	45%	55%	5%	0%	95%
Q 10. Does the NGO/CBO procure STI syndromic management medicines as per NACO/SACS guidelines?	40%	5%	55%	36%	36%	27%	73%**	0%	27%
Q 11. Has there been any change in the condom procurement process?	15%	15%	70%	45%	18%	36%	0%	5%	95%
Q 12. Has there been any change in the budget as per NACO/SACS guidelines?	5%	5%	90%	0%	18%	82%	0%	14%	86%

* See Section on Explanations for all cutoffs for Low/Medium/High; High means highly aligned

** Many of these TIs indicated that Avahan supply chain is still in place for medicines because they have a buffer stock in hand

	2012								
	FSW			MSM			Both		
	Low*	Medium	High	Low	Medium	High	Low*	Medium	High
Q 7. What is the present ratio of peer educators to HRG?*	0%	0%	100%	0%	36%	64%	0%	0%	100%
Q 8. What is the present ratio of outreach worker to HRG?**	0%	5%	95%	0%	27%	73%	0%	5%	95%

*NACO norm: Ratio of peer educators to HRG is 1: 60

**NACO norm: Ratio of outreach worker to HRG is 1: 250

	2012 Trainings			2012 Meetings		
Q 13 & 14. Have the following staffs received <u>training</u> for the transition OR	FSW	MSM	Both	FSW	MSM	Both

meetings on the transition as recommended by SACS?	None	1 staff	>1 staff	None	1 staff	>1 staff	None	1 staff	>1 staff	None	1 staff	>1 staff	None	1 staff	>1 staff	None	1 staff	>1 staff
TI guidelines	5%	0%	95%	0%	0%	100%	18%	41%	41%	25%	5%	70%	9%	0%	91%	59%	14%	27%
Program management	0%	10%	90%	9%	9%	82%	18%	36%	45%	35%	0%	65%	18%	0%	82%	64%	5%	32%
Outreach planning	5%	5%	90%	18%	0%	82%	23%	32%	45%	35%	0%	65%	18%	0%	82%	23%	32%	45%
Condom programing	10%	5%	85%	9%	0%	91%	32%	23%	45%	35%	5%	60%	9%	0%	91%	64%	5%	32%
Community mobilization	15%	0%	85%	18%	9%	73%	41%	9%	50%	40%	0%	60%	18%	0%	82%	68%	5%	27%
Communication	10%	0%	90%	18%	11%	0%	36%	32%	32%	40%	0%	60%	18%	0%	82%	68%	5%	27%
Counselling	10%	10%	80%	9%	9%	82%	59%	5%	36%	35%	0%	65%	18%	9%	73%	68%	9%	23%
STI management	0%	5%	95%	9%	0%	91%	45%	5%	50%	35%	0%	65%	18%	0%	82%	64%	5%	32%

* Percentages indicate the proportion of TIs in the state where 0, 1 or more than 1 type of staff attended the training for transition.

** Percentages show training that has been received. It does not count training that has been planned but has not yet taken place.

	2012 Trainings									2012 Meetings								
Q 13 & 14. Have the following staffs received training for the transition OR	FSW			MSM			Both			FSW			MSM			Both		
meetings on the transition as recommended by SACS?	Lowest	Average	Highest	Lowest	Average	Highest	Lowest	Average	Highest	Lowest	Average	Highest	Lowest	Average	Highest	Lowest	Average	Highest
PC/PD	2	7	12	2	5	7	0	5	8	0	5	8	0	6	8	0	3	8
Field Staff	0	6	12	1	4	6	0	2	8	0	5	8	0	5	8	0	2	8
Counsellor/ANM	0	6	12	3	6	8	0	2	8	0	5	8	0	6	8	0	2	8
Doctor	0	3	12	0	2	6	0	1	8	0	3	8	0	3	7	0	1	7
MIS Officer	2	6	12	0	4	6	0	2	8	0	5	8	0	5	8	0	2	8
ORW	0	6	12	2	5	8	0	4	12	0	5	8	2	6	8	0	3	12

* This indicates the lowest, average and highest numbers of types of trainings attended by each staff by state. Greater numbers indicate that staff in the respective category attended multiple types of trainings for the transition

	2012					
	FSW		MSM		Both	
Q 15. Does the NGO/CBO have a buffer in any of the following areas?	No	Yes	No	Yes	No	Yes
Medicines	50%	50%	64%	36%	14%	86%
Condoms	55%	45%	64%	36%	64%	36%
Funds	65%	35%	73%	27%	86%	14%

	2012											
	FSW				MSM				Both			
	No flexibility		Flexibility provided		No flexibility		Flexibility provided		No flexibility		Flexibility provided	
Q 16. Has there been any flexibility provided from NACO/SACS in the following areas?	Not important	Important	Not important	Important	Not important	Important	Not important	Important	Not important	Important	Not important	Important
Reporting	30%	50%	5%	15%	9%	82%	0%	9%	59%	32%	0%	9%
Staffing	15%	60%	0%	25%	9%	64%	0%	27%	23%	59%	9%	9%
Budgets	25%	60%	0%	15%	18%	82%	0%	0%	0%	82%	0%	18%
STI services	25%	45%	5%	25%	0%	45%	0%	55%	64%	18%	5%	14%
Condom programming	40%	40%	5%	15%	9%	55%	0%	36%	68%	14%	0%	18%
Linkages to ICTC	20%	35%	0%	45%	9%	55%	0%	36%	50%	23%	0%	27%

Section 2: TI Alignment - From Interviews

	2012								
	FSW			MSM			Both		
	Low	Medium	High	Low	Medium	High	Low	Medium	High
Q 1. Has there been any change in the Avahan method of micro-planning	30%	15%	55%	36%	9%	55%	4%	23%	73%
Q 2. Do all identified hotspots/halting places have condom outlets (TO, NTO, CVM)?	30%	55%	15%	27%	64%	9%	9%	82%	9%

	2012					
	FSW		MSM		Both	
Q 3. Does the NGO/CBO have a linkage with the following government services?	Most cases are referred	Most cases referred receive these services*	Most cases are referred	Most cases referred receive these services*	Most cases are referred	Most cases referred receive these services*
Government ICTC	95%	80%	100%	82%	91%	86%
ART centres	100%	75%	100%	82%	100%	86%
TB screening centres	95%	75%	100%	82%	95%	86%

* Percentages show NGO/CBOs where most cases are referred to government services AND those referred receive these services

	2012								
	FSW			MSM			Both		
Q 4. Which places do people go for STI services?	1st Choice (most preferred)	2nd Choice	3rd Choice	1st Choice (most preferred)	2nd Choice	3rd Choice	1st Choice (most preferred)	2nd Choice	3rd Choice

On-site clinic, Part time	20%	10%	15%	0%	0%	9%	32%	0%	0%
On-site clinic, Full time	35%	15%	20%	36%	0%	55%	64%	0%	14%
Government hospital clinic	15%	50%	25%	9%	82%	9%	0%	86%	13%
Preferred private practitioner	30%	25%	40%	55%	18%	27%	4%	14%	73%
None	0%	0%	0%	0%	0%	0%	0%	0%	0%

	2012								
	FSW			MSM			Both		
	Low	Medium	High	Low	Medium	High	Low	Medium	High
Q 5. Has the NGO/CBO been able to form groups at the community level?	0%	10%	90%	0%	18%	82%	4%	5%	91%
Q 6. Have community members at the hotspots formed crisis response committees?	0%	30%	70%	18%	0%	82%	0%	5%	95%

Section 3: TI Alignment - From CMIS Avahan

	2012											
	FSW				MSM				Both			
	Low	Medium	High	Don't know / NA	Low	Medium	High	Don't know / NA	Low	Medium	High	Don't know / NA
Q 1. What is the coverage of identified HRGs with regular contact (two contacts each month)?*	0%	0%	100%	0%	0%	18%	82%	0%	0%	5%	95%	0%
Q 2. What is the coverage of free condom supply among the identified HRG by NGO/CBO?**	0%	0%	20%	80%	0%	9%	0%	91%	0%	27%	55%	18%
Q 3. What percentage of HRGs who are referred actually visit the ICTC?***	30%	20%	50%	0%	27%	9%	64%	0%	64%	18%	18%	0%
Q 4. What is the coverage of syndromic management for HRGs with STI? ****	15%	5%	80%	0%	0%	0%	100%	0%	4%	9%	82%	5%

*NACO norm: 60% of identified HRGs have at least two contacts each month

**NACO norm: 100% of identified HRGs have access to free condoms

***NACO norm: 100% of HRGs who are referred should visit the ICTC

****NACO norm: 100% of HRGs with STI syndromes receive treatment

Transition Readiness Dashboard

From Round 1 & 2 Transition Readiness Assessment 2011 & 2012

Evaluative Assessment of Phase II of the Avahan Program: Transition and Influence Goals

9. Results of HRGs by Split TIs

	2012		2011	
	Not Split	Split	Not Split	Split
Did the TI Split due to transition?	26	27	25	2

	2012			
	Not Split		Split	
	NGO	CBO	NGO	CBO
Are you a NGO or CBO?	18	8	16	11

	2012					
	Not Split			Split		
	FSW	MSM	Both	FSW	MSM	Both
For whom do you provide services?	8	1	17	12	10	5

	2012							
	Not Split				Split			
	AP*	KR	TN	MH	AP	KR	TN	MH
In which state are you located?	12	2	7	5	4	15	0	8

*AP: Andra Pradesh, KR: Karnataka, TN: Tamil Nadu, MH: Maharashtra

Section 1: NGO/CBO Capacity

	2012					
	Not Split			Split		
	None	Societies Registration Act	States Trusts Acts	None	Societies Registration Act	States Trusts Acts
Q 1. What is the legal status of the organization? (mention the registration details under the societies / public trusts as applicable)	0%	77%	23%	0%	85%	15%

	2012			
	Not Split		Split	
	No	Yes	No	Yes
Q 1.1. Does the organization have a Foreign Contribution (Regulation) Act 1976 account?	23%	77%	19%	81%

	2012					
	Not Split			Split		
	Low*	Medium	High	Low	Medium	High
Q 2. Have the staff been informed about the transition?	0%	50%	50%	0%	11%	89%
Q 3. Does the NGO/CBO have a JAT (Joint Appraisal Team) score?	27%	46%	27%	37%	33%	30%
Q 4. Has there been communication about empanelment from SACS?	23%	15%	62%	7%	15%	78%
Q 5. Has there been any change in the reporting format, and are you sending any reports to SACS/District AIDS Control Societies?	15%	4%	81%	0%	0%	100%

Q 6. Has there been any change in the TI team structure, and are you following the SACS/NACO guidelines?	0%	8%	92%	0%	0%	100%
Q 9. Is the NGO/CBO following the STI syndromic management guideline of NACO?	4%	4%	92%	0%	22%	78%
Q 10. Does the NGO/CBO procure STI syndromic management medicines as per NACO/SACS guidelines?	65%**	0%	35%	41%	18%	41%
Q 11. Has there been any change in the condom procurement process?	11%	8%	81%	18%	15%	67%
Q 12. Has there been any change in the budget as per NACO/SACS guidelines?	0%	15%	85%	4%	7%	89%

* See Section on Explanations for all cutoffs for Low/Medium/High; High means highly aligned

** Many of these TIs indicated that Avahan supply chain is still in place for medicines because they have a buffer stock in hand

	2012					
	Not Split			Split		
	Low*	Medium	High	Low	Medium	High
Q 7. What is the present ratio of peer educators to HRG?*	0%	0%	100%	0%	15%	85%
Q 8. What is the present ratio of outreach worker to HRG?**	0%	4%	96%	0%	15%	85%

*NACO norm: Ratio of peer educators to HRG is 1: 60

**NACO norm: Ratio of outreach worker to HRG is 1: 250

	2012 Trainings						2012 Meetings					
Q 13 & 14. Have the following staffs received training for the transition OR meetings on the transition as recommended by SACS?	Not Split			Split			Not Split			Split		
	None	1 staff	>1 staff	None	1 staff	>1 staff	None	1 staff	>1 staff	None	1 staff	>1 staff
TI guidelines	15%	23%	62%	4%	11%	85%	50%	4%	46%	19%	4%	78%
Program management	12%	19%	69%	7%	22%	70%	50%	8%	42%	22%	7%	70%
Outreach planning	19%	19%	62%	11%	11%	78%	54%	0%	46%	33%	4%	63%
Condom programing	23%	15%	62%	15%	7%	78%	54%	0%	46%	33%	4%	63%
Community mobilization	31%	4%	65%	22%	7%	70%	54%	4%	42%	41%	0%	59%
Communication	31%	19%	50%	15%	7%	78%	54%	4%	42%	41%	0%	59%
Counselling	42%	4%	54%	19%	11%	70%	54%	8%	38%	37%	4%	59%
STI management	27%	4%	69%	15%	4%	81%	54%	0%	46%	33%	4%	63%

* Percentages indicate the proportion of TIs in the state where 0, 1 or more than 1 type of staff attended the training for transition.

** Percentages show training that has been received. It does not count training that has been planned but has not yet taken place.

	2012 Trainings						2012 Meetings					
Q 13 & 14. Have the following staffs received training for the transition OR meetings on the transition as recommended by SACS?	Not Split			Split			Not Split			Split		
	Lowest	Average	Highest	Lowest	Average	Highest	Lowest	Average	Highest	Lowest	Average	Highest
PC/PD	0	6	12	0	6	8	0	3	8	0	5	8
Field Staff	0	4	12	0	4	7	0	3	8	0	4	8
Counsellor/ANM	0	4	12	0	5	8	0	3	8	0	5	8
Doctor	0	2	12	0	2	7	0	2	8	0	2	7
MIS Officer	0	4	12	0	4	7	0	3	8	0	4	8
ORW	0	5	12	0	5	8	0	4	12	0	4	8

* This indicates the lowest, average and highest numbers of types of trainings attended by each staff by state. Greater numbers indicate that staff in the respective category attended multiple types of trainings for the transition

	2012			
	Not Split		Split	
Q 15. Does the NGO/CBO have a buffer in any of the following areas?	No	Yes	No	Yes
Medicines	23%	77%	52%	48%

Condoms	58%	42%	63%	37%
Funds	73%	27%	78%	22%

	2012							
	Not Split				Split			
	No flexibility		Flexibility provided		No flexibility		Flexibility provided	
Q 16. Has there been any flexibility provided from NACO/SACS in the following areas?	Not important	Important	Not important	Important	Not important	Important	Not important	Important
Reporting	58%	38%	0%	4%	19%	59%	4%	19%
Staffing	23%	62%	4%	12%	11%	59%	4%	26%
Budgets	8%	73%	0%	19%	19%	74%	0%	7%
STI services	50%	27%	0%	23%	22%	41%	7%	30%
Condom programming	62%	27%	0%	12%	30%	37%	4%	30%
Linkages to ICTC	42%	27%	0%	31%	19%	41%	0%	41%

Section 2: TI Alignment - From Interviews

	2012					
	Not Split			Split		
	Low	Medium	High	Low	Medium	High
Q 1. Has there been any change in the Avahan method of micro-planning	12%	23%	65%	30%	11%	59%
Q 2. Do all identified hotspots/halting places have condom outlets (TO, NTO, CVM)?	23%	62%	15%	19%	74%	7%

	2012			
	Not Split		Split	
Q 3. Does the NGO/CBO have a linkage with the following government services?	Most cases are referred	Most cases referred receive these services*	Most cases are referred	Most cases referred receive these services*
Government ICTC	88%	73%	100%	93%
ART centres	100%	69%	100%	93%
TB screening centres	92%	69%	100%	93%

* Percentages show NGO/CBOs where most cases are referred to government services AND those referred receive these services

	2012					
	Not Split			Split		
Q 4. Which places do people go for STI services?	1st Choice (most preferred)	2nd Choice	3rd Choice	1st Choice (most preferred)	2nd Choice	3rd Choice
On-site clinic, Part time	31%	0%	4%	11%	7%	11%
On-site clinic, Full time	57%	8%	8%	37%	4%	41%
Government hospital clinic	8%	73%	15%	7%	70%	18%
Preferred private practitioner	4%	19%	73%	45%	19%	30%
None	0%	0%	0%	0%	0%	0%

	2012					
	Not Split			Split		
	Low	Medium	High	Low	Medium	High
Q 5. Has the NGO/CBO been able to form groups at the community level?	4%	8%	88%	0%	11%	89%
Q 6. Have community members at the hotspots formed crisis response committees?	0%	19%	81%	7%	8%	85%

Section 3: TI Alignment - From CMIS Avahan

	2012							
	Not Split				Split			
	Low	Medium	High	Don't know / NA	Low	Medium	High	Don't know
Q 1. What is the coverage of identified HRGs with regular contact (two contacts each month)?*	0%	8%	92%	0%	0%	4%	96%	0%
Q 2. What is the coverage of free condom supply among the identified HRG by NGO/CBO?**	0%	15%	54%	31%	0%	11%	7%	82%
Q 3. What percentage of HRGs who are referred actually visit the ICTC?***	54%	19%	27%	0%	33%	15%	52%	0%
Q 4. What is the coverage of syndromic management for HRGs with STI? ****	12%	19%	69%	0%	33%	15%	52%	0%

*NACO norm: 60% of identified HRGs have at least two contacts each month
**NACO norm: 100% of identified HRGs have access to free condoms
***NACO norm: 100% of HRGs who are referred should visit the ICTC
****NACO norm: 100% of HRGs with STI syndromes receive treatment

Transition Readiness Dashboard

From Round 1 & 2 Transition Readiness Assessment 2011 & 2012
Evaluative Assessment of Phase II of the Avahan Program: Transition and Influence Goals

10. Results of HRGs by Urban/Rural/Both

	2012			2011		
	Urban	Rural	Both	Urban	Rural	Both
Is the TI located in an urban area, rural area or both?	28	11	14	16	4	7

	2012					
	Urban		Rural		Both	
	NGO	CBO	NGO	CBO	NGO	CBO
Are you a NGO or CBO?	16	12	9	2	9	5

	2012											
	Urban				Rural				Both			
	AP	KR	TN	MH	AP	KR	TN	MH	AP	KR	TN	MH
In which state are you located?	7	15	0	6	4	2	1	4	5	0	6	3

*AP: Andra Pradesh, KR: Karnataka, TN: Tamil Nadu, MH: Maharashtra

Section 1: NGO/CBO Capacity

	2012								
	Urban			Rural			Both		
	None	Societies Registration Act	States Trusts Acts	None	Societies Registration Act	States Trusts Acts	None	Societies Registration Act	States Trusts Acts
Q 1. What is the legal status of the organization? (mention the registration details under the societies / public trusts as applicable)	0%	89%	11%	0%	73%	27%	0%	71%	29%

	2012					
	Urban		Rural		Both	
	No	Yes	No	Yes	No	Yes
Q 1.1. Does the organization have a Foreign Contribution (Regulation) Act 1976 account?	29%	71%	0%	100%	21%	79%

	2012								
	Urban			Rural			Both		
	Low*	Medium	High	Low	Medium	High	Low*	Medium	High
Q 2. Have the staff been informed about the transition?	0%	25%	75%	0%	27%	73%	0%	43%	57%
Q 3. Does the NGO/CBO have a JAT (Joint Appraisal Team) score?	39%	43%	18%	18%	27%	55%	29%	43%	29%
Q 4. Has there been communication about empanelment from SACS?	11%	14%	75%	9%	18%	73%	29%	14%	57%
Q 5. Has there been any change in the reporting format, and are you sending any reports to SACS/District AIDS Control Societies?	0%	0%	100%	9%	0%	91%	21%	7%	71%
Q 6. Has there been any change in the TI team structure, and are you following the SACS/NACO guidelines?	0%	7%	93%	0%	0%	100%	0%	0%	100%
Q 9. Is the NGO/CBO following the STI syndromic management guideline of NACO?	0%	18%	82%	0%	18%	82%	7%	0%	93%
Q 10. Does the NGO/CBO procure STI syndromic management medicines as per NACO/SACS guidelines?	43%	18%	39%	82%**	0%	18%	50%	0%	50%
Q 11. Has there been any change in the condom procurement process?	18%	18%	64%	18%	0%	82%	7%	7%	86%
Q 12. Has there been any change in the budget as per NACO/SACS guidelines?	4%	7%	89%	0%	9%	91%	0%	21%	79%

* See Section on Explanations for all cutoffs for Low/Medium/High; High means highly aligned
** Many of these TIs indicated that Avahan supply chain is still in place for medicines because they have a buffer stock in hand

	2012								
	Urban			Rural			Both		
	Low*	Medium	High	Low	Medium	High	Low*	Medium	High
Q 7. What is the present ratio of peer educators to HRG?*	0%	14%	86%	0%	0%	100%	0%	0%	100%
Q 8. What is the present ratio of outreach worker to HRG?**	0%	7%	93%	0%	18%	82%	0%	7%	93%

*NACO norm: Ratio of peer educators to HRG is 1: 60
**NACO norm: Ratio of outreach worker to HRG is 1: 250

	2012 Trainings			2012 Meetings		
Q 13 & 14. Have the following staffs received <u>training</u> for the transition OR	Urban	Rural	Both	Urban	Rural	Both

meetings on the transition as recommended by SACS?	None	1 staff	>1 staff	None	1 staff	>1 staff	None	1 staff	>1 staff	None	1 staff	>1 staff	None	1 staff	>1 staff	None	1 staff	>1 staff
TI guidelines	4%	18%	79%	18%	18%	64%	14%	14%	71%	36%	4%	61%	27%	9%	64%	36%	0%	64%
Program management	7%	25%	68%	9%	18%	73%	14%	14%	71%	36%	7%	57%	36%	9%	55%	36%	7%	57%
Outreach planning	18%	14%	68%	9%	18%	73%	14%	14%	71%	50%	0%	50%	36%	9%	55%	36%	0%	64%
Condom programing	21%	14%	64%	18%	9%	73%	14%	7%	79%	50%	0%	50%	36%	9%	55%	36%	0%	64%
Community mobilization	32%	7%	61%	18%	9%	73%	21%	0%	79%	54%	0%	46%	45%	0%	55%	36%	7%	57%
Communication	21%	11%	68%	36%	9%	0%	14%	21%	64%	54%	0%	46%	45%	0%	55%	36%	7%	57%
Counselling	32%	11%	57%	27%	9%	64%	29%	0%	71%	50%	4%	46%	45%	0%	55%	36%	14%	50%
STI management	18%	7%	75%	27%	0%	73%	21%	0%	79%	50%	0%	50%	36%	9%	55%	36%	0%	64%

* Percentages indicate the proportion of TIs in the state where 0, 1 or more than 1 type of staff attended the training for transition.

** Percentages show training that has been received. It does not count training that has been planned but has not yet taken place.

	2012 Trainings									2012 Meetings								
Q 13 & 14. Have the following staffs received training for the transition OR	Urban			Rural			Both			Urban			Rural			Both		
meetings on the transition as recommended by SACS?	Lowest	Average	Highest	Lowest	Average	Highest	Lowest	Average	Highest	Lowest	Average	Highest	Lowest	Average	Highest	Lowest	Average	Highest
PC/PD	0	6	12	0	6	8	0	6	8	0	4	8	0	5	8	0	5	8
Field Staff	0	4	12	0	4	8	0	4	8	0	3	8	0	4	8	0	4	8
Counsellor/ANM	0	5	12	0	4	8	0	4	8	0	4	8	0	4	8	0	4	8
Doctor	0	2	12	0	3	8	0	3	8	0	2	8	0	3	8	0	2	7
MIS Officer	0	4	12	0	4	8	0	4	8	0	3	8	0	4	8	0	4	8
ORW	0	4	12	0	4	8	0	4	12	0	3	8	2	4	8	0	4	12

* This indicates the lowest, average and highest numbers of types of trainings attended by each staff by state. Greater numbers indicate that staff in the respective category attended multiple types of trainings for the transition

	2012					
	Urban		Rural		Both	
Q 15. Does the NGO/CBO have a buffer in any of the following areas?	No	Yes	No	Yes	No	Yes
Medicines	64%	36%	9%	91%	7%	93%
Condoms	79%	21%	36%	64%	43%	57%
Funds	89%	11%	55%	45%	64%	36%

	2012											
	Urban				Rural				Both			
	No flexibility		Flexibility provided		No flexibility		Flexibility provided		No flexibility		Flexibility provided	
Q 16. Has there been any flexibility provided from NACO/SACS in the following areas?	Not important	Important	Not important	Important	Not important	Important	Not important	Important	Not important	Important	Not important	Important
Reporting	32%	54%	0%	14%	45%	36%	9%	9%	43%	50%	0%	7%
Staffing	11%	64%	4%	21%	9%	64%	9%	18%	36%	50%	0%	14%
Budgets	21%	71%	0%	7%	0%	82%	0%	18%	7%	71%	0%	21%
STI services	32%	29%	7%	32%	36%	36%	0%	27%	43%	43%	0%	14%
Condom programming	43%	29%	4%	25%	45%	36%	0%	18%	50%	36%	0%	14%
Linkages to ICTC	32%	29%	0%	39%	27%	55%	0%	18%	29%	29%	0%	43%

Section 2: TI Alignment - From Interviews

	2012								
	Urban			Rural			Both		
	Low	Medium	High	Low	Medium	High	Low	Medium	High
Q 1. Has there been any change in the Avahan method of micro-planning	36%	21%	43%	9%	9%	82%	0%	14%	86%
Q 2. Do all identified hotspots/halting places have condom outlets (TO, NTO, CVM)?	25%	61%	14%	9%	82%	9%	21%	71%	7%

	2012					
	Urban		Rural		Both	
Q 3. Does the NGO/CBO have a linkage with the following government services?	Most cases are referred	Most cases referred receive these services*	Most cases are referred	Most cases referred receive these services*	Most cases are referred	Most cases referred receive these services*
Government ICTC	100%	93%	100%	73%	78%	71%
ART centres	100%	93%	100%	64%	100%	71%
TB screening centres	100%	93%	100%	64%	95%	71%

* Percentages show NGO/CBOs where most cases are referred to government services AND those referred receive these services

	2012								
	Urban			Rural			Both		
Q 4. Which places do people go for STI services?	1st Choice (most preferred)	2nd Choice	3rd Choice	1st Choice (most preferred)	2nd Choice	3rd Choice	1st Choice (most preferred)	2nd Choice	3rd Choice

On-site clinic, Part time	11%	0%	7%	18%	9%	18%	43%	7%	0%
On-site clinic, Full time	54%	7%	29%	27%	9%	18%	50%	0%	21%
Government hospital clinic	7%	71%	14%	18%	82%	0%	0%	64%	36%
Preferred private practitioner	29%	21%	50%	36%	0%	64%	7%	29%	43%
None	0%	0%	0%	0%	0%	0%	0%	0%	0%

	2012								
	Urban			Rural			Both		
	Low	Medium	High	Low	Medium	High	Low	Medium	High
Q 5. Has the NGO/CBO been able to form groups at the community level?	0%	14%	86%	0%	9%	91%	7%	0%	93%
Q 6. Have community members at the hotspots formed crisis response committees?	7%	14%	79%	0%	9%	91%	0%	14%	86%

Section 3: TI Alignment - From CMIS Avahan

	2012											
	Urban				Rural				Both			
	Low	Medium	High	Don't know / NA	Low	Medium	High	Don't know / NA	Low	Medium	High	Don't know / NA
Q 1. What is the coverage of identified HRGs with regular contact (two contacts each month)?*	0%	4%	96%	0%	0%	0%	100%	0%	0%	7%	93%	0%
Q 2. What is the coverage of free condom supply among the identified HRG by NGO/CBO?**	0%	11%	21%	68%	0%	9%	27%	64%	0%	29%	50%	21%
Q 3. What percentage of HRGs who are referred actually visit the ICTC?***	39%	18%	43%	0%	55%	0%	45%	0%	43%	29%	29%	0%
Q 4. What is the coverage of syndromic management for HRGs with STI? ****	32%	14%	54%	0%	9%	36%	55%	0%	14%	7%	79%	0%

*NACO norm: 60% of identified HRGs have at least two contacts each month
**NACO norm: 100% of identified HRGs have access to free condoms
***NACO norm: 100% of HRGs who are referred should visit the ICTC
****NACO norm: 100% of HRGs with STI syndromes receive treatment

Transition Readiness Dashboard

From Round 1 & 2 Transition Readiness Assessment 2011 & 2012

Evaluative Assessment of Phase II of the Avahan Program: Transition and Influence Goals

11. Results of HRGs by April vs. later Transition

*Later includes TIs that transitioned from July 2012 onwards (including a TI not yet transitioned as of Jan 2013)

	2012	
	April	Later
When did the TI transition?	43	10

	2012			
	April		Later	
	NGO	CBO	NGO	CBO
Are you a NGO or CBO?	31	12	3	7

	2012					
	April			Later		
	FSW	MSM	Both	FSW	MSM	Both
For whom do you provide services?	18	9	16	2	2	6

	2012							
	April				Later			
	AP*	KR	TN	MH	AP	KR	TN	MH
In which state are you located?	13	13	4	13	3	4	3	0

*AP: Andra Pradesh, KR: Karnataka, TN: Tamil Nadu, MH: Maharashtra

Section 1: NGO/CBO Capacity

	2012					
	April			Later		
	None	Societies Registration Act	States Trusts Acts	None	Societies Registration Act	States Trusts Acts
Q 1. What is the legal status of the organization? (mention the registration details under the societies / public trusts as applicable)	0%	79%	21%	0%	90%	10%

	2012			
	April		Later	
	No	Yes	No	Yes
Q 1.1. Does the organization have a Foreign Contribution (Regulation) Act 1976 account?	21%	79%	20%	80%

	2012					
	April			Later		
	Low*	Medium	High	Low	Medium	High
Q 2. Have the staff been informed about the transition?	0%	28%	72%	0%	40%	60%
Q 3. Does the NGO/CBO have a JAT (Joint Appraisal Team) score?	30%	37%	33%	40%	50%	10%
Q 4. Has there been communication about empanelment from SACS?	14%	12%	74%	20%	30%	50%

Q 5. Has there been any change in the reporting format, and are you sending any reports to SACS/District AIDS Control Societies?	9%	0%	91%	0%	10%	90%
Q 6. Has there been any change in the TI team structure, and are you following the SACS/NACO guidelines?	0%	5%	95%	0%	0%	100%
Q 9. Is the NGO/CBO following the STI syndromic management guideline of NACO?	0%	14%	86%	10%	10%	80%
Q 10. Does the NGO/CBO procure STI syndromic management medicines as per NACO/SACS guidelines?	56%	9%	35%	40%	10%	50%
Q 11. Has there been any change in the condom procurement process?	19%	9%	72%	0%	20%	80%
Q 12. Has there been any change in the budget as per NACO/SACS guidelines?	2%	12%	86%	0%	10%	90%

* See Section on Explanations for all cutoffs for Low/Medium/High; High means highly aligned

** Many of these TIs indicated that Avahan supply chain is still in place for medicines because they have a buffer stock in hand

	2012					
	April			Later		
	Low*	Medium	High	Low	Medium	High
Q 7. What is the present ratio of peer educators to HRG?*	0%	5%	95%	0%	20%	80%
Q 8. What is the present ratio of outreach worker to HRG?**	0%	12%	88%	0%	0%	100%

*NACO norm: Ratio of peer educators to HRG is 1: 60

**NACO norm: Ratio of outreach worker to HRG is 1: 250

	2012 Trainings						2012 Meetings					
Q 13 & 14. Have the following staffs received training for the transition OR meetings on the transition as recommended by SACS?	April			Later			April			Later		
	None	1 staff	>1 staff	None	1 staff	>1 staff	None	1 staff	>1 staff	None	1 staff	>1 staff
TI guidelines	9%	19%	72%	10%	10%	80%	35%	5%	60%	30%	0%	70%
Program management	9%	14%	77%	10%	0%	90%	37%	7%	56%	30%	10%	60%
Outreach planning	14%	19%	67%	20%	0%	80%	44%	2%	53%	40%	0%	60%
Condom programing	19%	14%	67%	20%	0%	80%	44%	2%	53%	40%	0%	60%
Community mobilization	28%	7%	65%	20%	0%	80%	49%	0%	51%	40%	10%	50%
Communication	23%	16%	60%	5%	0%	95%	49%	2%	49%	40%	0%	60%
Counselling	33%	9%	58%	20%	0%	80%	47%	5%	49%	40%	10%	50%
STI management	23%	5%	72%	10%	0%	90%	44%	2%	53%	40%	0%	60%

* Percentages indicate the proportion of TIs in the state where 0, 1 or more than 1 type of staff attended the training for transition.

** Percentages show training that has been received. It does not count training that has been planned but has not yet taken place.

	2012 Trainings						2012 Meetings					
Q 13 & 14. Have the following staffs received training for the transition OR meetings on the transition as recommended by SACS?	April			Later			April			Later		
	Lowest	Average	Highest	Lowest	Average	Highest	Lowest	Average	Highest	Lowest	Average	Highest
PC/PD	0	6	12	0	6	8	0	4	8	0	5	8
Field Staff	0	4	12	0	4	7	0	3	8	0	4	8
Counsellor/ANM	0	5	12	0	4	8	0	4	8	0	4	8
Doctor	0	2	12	0	2	5	0	2	8	0	3	7
MIS Officer	0	4	12	0	4	7	0	3	8	0	3	8
ORW	0	4	12	0	6	12	0	4	12	0	4	8

* This indicates the lowest, average and highest numbers of types of trainings attended by each staff by state. Greater numbers indicate that staff in the respective category attended multiple types of trainings for the transition

2012	
April	Later

Q 15. Does the NGO/CBO have a buffer in any of the following areas?	No	Yes	No	Yes
Medicines	35%	65%	50%	50%
Condoms	58%	42%	70%	30%
Funds	74%	26%	80%	20%

	2012							
	April				Later			
	No flexibility		Flexibility provided		No flexibility		Flexibility provided	
Q 16. Has there been any flexibility provided from NACO/SACS in the following areas?	Not important	Important	Not important	Important	Not important	Important	Not important	Important
Reporting	40%	47%	2%	12%	30%	60%	0%	10%
Staffing	16%	60%	5%	19%	20%	60%	0%	20%
Budgets	14%	74%	0%	12%	10%	70%	0%	20%
STI services	37%	35%	5%	23%	30%	30%	0%	40%
Condom programming	49%	35%	2%	14%	30%	20%	0%	50%
Linkages to ICTC	30%	37%	0%	33%	30%	20%	0%	50%

Section 2: TI Alignment - From Interviews

	2012					
	April			Later		
	Low	Medium	High	Low	Medium	High
Q 1. Has there been any change in the Avahan method of micro-planning	23%	19%	58%	10%	10%	80%
Q 2. Do all identified hotspots/halting places have condom outlets (TO, NTO, CVM)?	19%	70%	12%	30%	60%	10%

	2012			
	April		Later	
Q 3. Does the NGO/CBO have a linkage with the following government services?	Most cases are referred	Most cases referred receive these services*	Most cases are referred	Most cases referred receive these services*
Government ICTC	95%	81%	90%	90%
ART centres	100%	79%	100%	90%
TB screening centres	95%	79%	100%	90%

* Percentages show NGO/CBOs where most cases are referred to government services AND those referred receive these services

	2012					
	April			Later		
Q 4. Which places do people go for STI services?	1st Choice (most preferred)	2nd Choice	3rd Choice	1st Choice (most preferred)	2nd Choice	3rd Choice
On-site clinic, Part time	23%	5%	9%	10%	0%	0%
On-site clinic, Full time	37%	7%	30%	90%	0%	0%
Government hospital clinic	9%	74%	12%	0%	60%	40%
Preferred private practitioner	30%	14%	49%	0%	40%	60%
None	0%	0%	0%	0%	0%	0%

	2012					
	April			Later		
	Low	Medium	High	Low	Medium	High
Q 5. Has the NGO/CBO been able to form groups at the community level?	0%	9%	91%	10%	10%	80%

Q 6. Have community members at the hotspots formed crisis response committees?	5%	16%	79%	0%	0%	100%

Section 3: TI Alignment - From CMIS Avahan

	2012							
	April				Later			
	Low	Medium	High	Don't know / NA	Low	Medium	High	Don't know
Q 1. What is the coverage of identified HRGs with regular contact (two contacts each month)?*	0%	2%	98%	0%	0%	10%	90%	0%
Q 2. What is the coverage of free condom supply among the identified HRG by NGO/CBO?**	0%	12%	28%	60%	0%	30%	40%	30%
Q 3. What percentage of HRGs who are referred actually visit the ICTC?***	49%	16%	35%	0%	20%	20%	60%	0%
Q 4. What is the coverage of syndromic management for HRGs with STI? ****	21%	19%	60%	0%	30%	10%	60%	0%

*NACO norm: 60% of identified HRGs have at least two contacts each month
**NACO norm: 100% of identified HRGs have access to free condoms
***NACO norm: 100% of HRGs who are referred should visit the ICTC
****NACO norm: 100% of HRGs with STI syndromes receive treatment

Transition Readiness Dashboard

From Round 1 & 2 Transition Readiness Assessment 2011 & 2012
Evaluative Assessment of Phase II of the Avahan Program: Transition and Influence Goals

12. Explanations of Low/Medium/High Categories

This study applied a questionnaire for transition, which highlights the key issues in aligning Avahan HRG TI programs to NACO guidelines. The interviews asked about elements of transition preparedness in terms of NGO/CBO capacity and TI alignment. This questionnaire was developed based upon field visits conducted, pre-tests, administrative record reviews, as well as documentation on NACO standards for transitioning TIs. Below are the cutoffs used in the survey for determining the Low/Middle/High levels of transition readiness.

Section 1: NGO/CBO Capacity

	HRGs		
	**Low**	**Medium**	**High**
Q 2. Have the staff been informed about the transition?	Not informed	Has been discussed	Transition plans have been developed with staff inputs
Q 3. Does the NGO/CBO have a JAT (Joint Appraisal Team) score?*	JAT not taken place	JAT completed but scores not available	JAT score available
Q 4. Has there been communication about empanelment from SACS?	Process not initiated	Process initiated	NGO/CBO empanelled
Q 5. Has there been any change in the reporting format, and are you sending any reports to SACS/District AIDS Control Societies?	No change in reporting format	SACS formats discussed but not all introduced	Following all SACS formats
Q 6. Has there been any change in the TI team structure, and are you following the SACS/NACO guidelines?	No change in team structure	Some changes were introduced	Following SACS TI structure
Q 7. What is the present ratio of peer educators to HRG?	Ratio was not previously measured	Ratio is measured and approaching that of SACS	Following the SACS ratio
Q 8. What is the present ratio of outreach worker to HRG?	Ratio was not previously measured	Ratio is measured and approaching that of SACS	Following the SACS ratio
Q 9. Is the NGO/CBO following the STI syndromic management guideline of NACO?	Avahan guidelines are still in place	Some changes are made according to NACO guidelines	Following STI syndromic management guidelines of NACO

Q 10. Does the NGO/CBO procure STI syndromic management medicines as per NACO/SACS guidelines?	Avahan supply chain is still in place	Some changes are made in the procurement processes as suggested by NACO/SACS	STI syndromic management medicines are procured as per NACO/SACS guidelines
Q 11. Has there been any change in the condom procurement process?	process not initiated	Some changes are made in the condom procurement processes	All condom procurement is done through channels suggested by SACS
Q 12. Has there been any change in the budget as per NACO/SACS guidelines?	No change, still following Avahan budget	Some changes were made to the budget	Following NACO/SACS budget guidelines

Section 2: TI Alignment - From Interviews

	HRGs		
	**Low**	**Medium**	**High**
Q 1. Has there been any change in the Avahan method of micro-planning	Avahan method of micro-planning is still in place	Some changes are made to micro-planning	Micro-plan follows NACO guidelines and is updated every quarter
Q 2. Do all identified hotspots within the TSL have condom outlets (TO, NTO, CVM)?	None have condom outlets	Some hotspots within the TSL have condom outlets	All hotspots within the TSL have condom outlets
Q 5. Has the NGO/CBO been able to form groups at the community level?	Groups have not been formed	Group formation in process	Groups have been formed
Q 6. Have community members at the hotspots formed crisis response committees?	No committees formed	Committees formed, but less than 30% of members meet every month	Committees formed, and 30% or more members meet every month

Section 3: TI Alignment - From CMIS Avahan

	HRGs		
	**Low**	**Medium**	**High**

Q 1. What is the coverage of identified HRGs with regular contact (two contacts each month)?	Some HRGs contacted in last month (<30%)	Over 30% contacted in last month (<60%)	60% or more contacted in the last month
Q 2. What is the coverage of free condom supply among the identified HRG by NGO/CBO?	No free supply of condoms	Some free supply of condoms (1-99%)	Free supply of condoms are fully available
Q 3. What percentage of HRGs who are referred actually visit the ICTC?	Poor coverage (<50%)	Over 50% of HRGs referred actually visit the ICTC (<100%)	100% of HRGs referred actually visit the ICTC
Q 4. What is the coverage of syndromic management for HRGs with STI?	Poor coverage (<50%)	Over 50% of HRGs with STI syndromes receive treatment (<100%)	100% of HRGs with STI syndromes receive treatment
