# Supplementary material for: Transitioning a Large Scale HIV/AIDS Prevention Program to Local Stakeholders: Findings from the Avahan Transition Evaluation
Source: PLoS One. 2015 Sep 1;10(9):e0136177. doi: 10.1371/journal.pone.0136177 (PMC4556643; doi:10.1371/journal.pone.0136177)
Supplement: S2 Table — (PDF) [file pone.0136177.s002.pdf]

WP 4: Institutionalization Study

Quantitative Results from Round 1

Study Overview

This study component focused on how well Avahan characteristics have become incorporated or ‘institutionalized’ by the Targeted Interventions (TIs) for high risk groups (HRGs) post-transition, from Avahan management and funding to that of State AIDS Control Societies (SACS). Round 1 of the survey was conducted between August-November 2012 among Project Directors (PD) or Coordinators (PC) of all TIs that transitioned in Spring 2011 (n=28), across 4 states.

	Inst. Round 1	TR Round 1*		
No. of TIs	28	27		
	Andra Pradesh	Karnataka	Tamil Nadu	Maharashtra
In which state are you located?	11	5	4	8
	FSW	MSM	Both	
For whom do you provide services?	13	4	11	
	NGO	CBO		
Are you a NGO or CBO?	19	9		
	Not Split	Split		
Did the TI Split due to transition?	15	13		

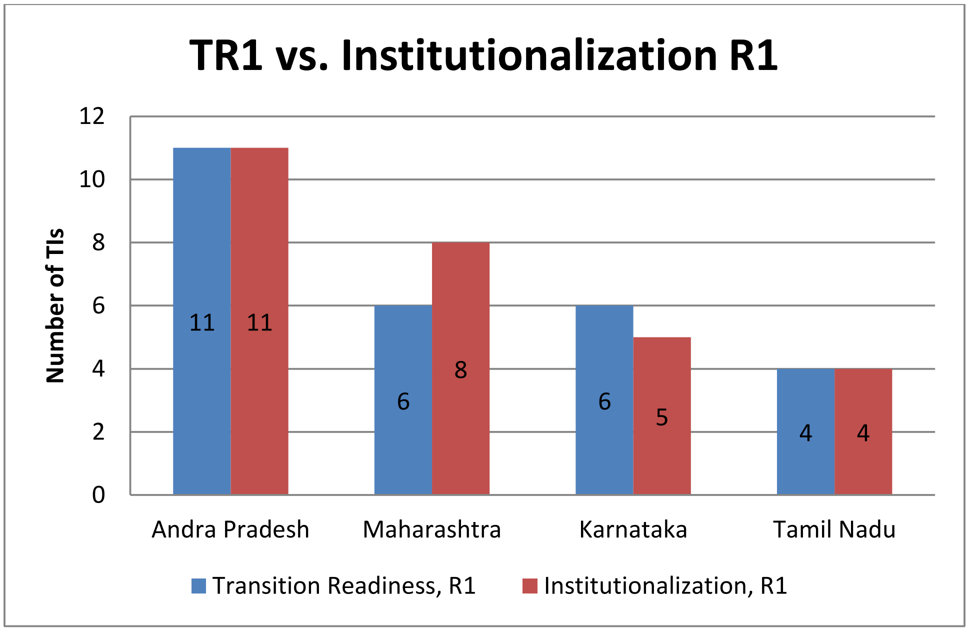

Transition Timing & Preparation

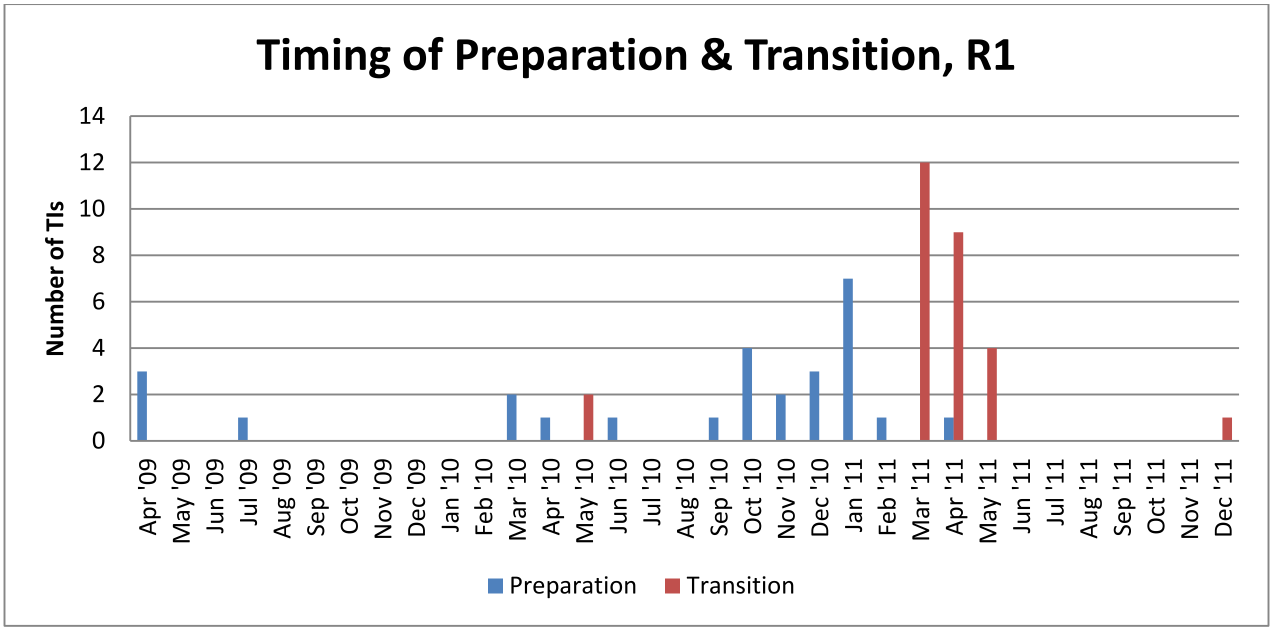

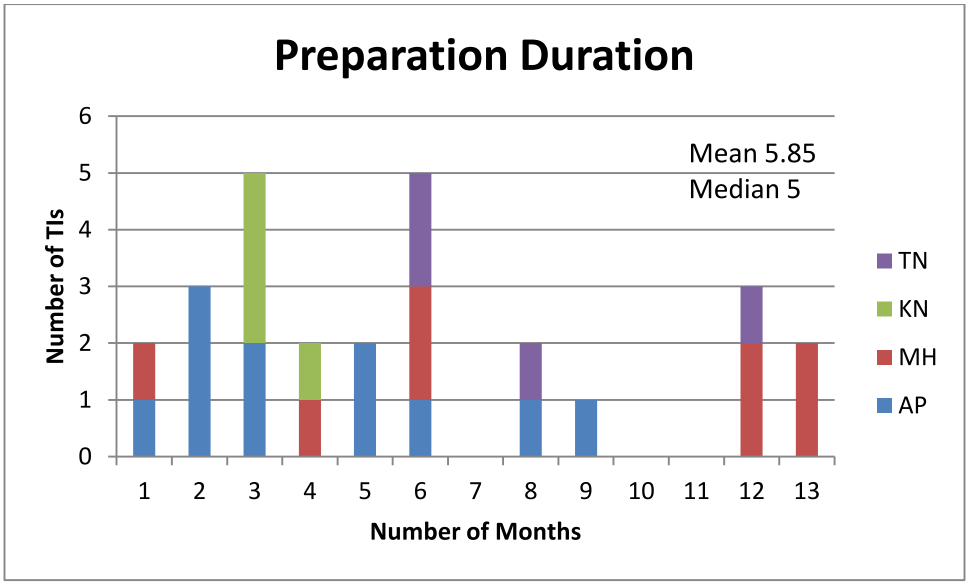

Transition Experience

Perceptions	Strongly Agree	Agree	Neutral	Disagree	Strongly Disagree	No Response
1. Overall, the transition experience went smoothly	36%	43%	11%	7%	0%	4%
2. The overall program has changed significantly as compared to pre-transition	36%	39%	0%	18%	4%	4%

3. SACS has the same or a higher level of commitment toward the program as compared to Avahan	18%	64%	11%	4%	0%	4%
4. The NGO/CBO has received support from the SLP after the transition	39%	50%	0%	4%	4%	4%

Data use & Supervision

[AC1: Active use of data at all levels for planning and regular review of program delivery]

	Regularly	Sometimes	Never	No Response
B.1 Does your NGO/CBO actively use data for program planning	89%	0%	0%	11%
	Yes	No		No Response
B.2 * Has the use of data for planning changed due to the transition?	57%	39%		4%
	The better	The worse	Made no difference	No Response
a. If YES, was this a change for?	88%	0%	13%	0%
	SACS	NGO/CBO	Other	No Response
b. If YES, who brought about this change?	63%	38%	0%	0%

By State (# of TIs)	Andra Pradesh	Karnataka	Tamil Nadu	Maharashtra
B.1 Does your NGO/CBO actively use data for program planning: Regularly	11/11	5/5	4/4	5/8
B.2 * Has the use of data for planning changed due to the transition: Yes	5/11	3/5	4/4	4/8
a. If YES, was this a change for: The better	5/5	3/3	4/4	2/4
b. If YES, who brought about this change: SACS	4/5	0/3	2/4	4/4

Perceptions	Strongly Agree	Agree	Neutral	Disagree	Strongly Disagree	No Response
5. The NGO/CBO actively uses data for program planning [AC1]	68%	32%	0%	0%	0%	0%

	Regularly	Sometimes	Never	No Response
B.3 Does your NGO/CBO actively use data to monitor progress in the program?	100%	0%	0%	0%
	Yes	No		No Response
B.4 * Has the use of data to monitor progress changed due to the transition?	68%	29%		4%
	The better	The worse	Made no	No Response

			difference	
a. If YES, was this a change for?	79%	11%	11%	0%
	SACS	NGO/CBO	Other	No Response
b. If YES, who brought about this change?	95%	5%	0%	0%

By State	Andra Pradesh	Karnataka	Tamil Nadu	Maharashtra
B.3 Does your NGO/CBO actively use data to monitor progress in the program: Regularly	11/11	5/5	4/4	8/8
B.4 * Has the use of data to monitor progress changed due to the transition: Yes	11/11	2/5	4/4	2/8
a. If YES, was this a change for: The better	7/11	2/2	4/4	2/2
b. If YES, who brought about this change: SACS	10/11	2/2	4/4	2/2

Perceptions	Strongly Agree	Agree	Neutral	Disagree	Strongly Disagree	No Response
6. The NGO/CBO actively uses data to monitor program progress [AC1]	64%	36%	0%	0%	0%	0%

[AC2: Pictorial micro-planning tool for peer outreach workers]

	Regularly	Sometimes	Never	No Response
B.5 Do peer outreach workers use pictorial micro-planning to facilitate their mapping of most at risk populations?	96%	4%	0%	0%
	Yes	No		No Response
B.6 * Has the use of pictorial micro-planning tool changed due to the transition?	46%	50%		4%
	The better	The worse	Made no difference	No Response
a. If YES, was this a change for?	92%	0%	8%	0%
	SACS	NGO/CBO	Other	No Response
b. If YES, who brought about this change?	85%	0%	15%	0%

By State	Andra Pradesh	Karnataka	Tamil Nadu	Maharashtra
B.5 Do peer outreach workers use pictorial micro-planning to facilitate their mapping of most at risk populations: Regularly	11/11	5/5	4/4	7/8
B.6 * Has the use of pictorial micro-planning tool changed due to the transition: Yes	5/11	2/5	4/4	2/8
a. If YES, was this a change for: The better	4/5	2/2	4/4	2/2
b. If YES, who brought about this change: SACS	5/5	0/2	4/4	2/2

Perceptions	Strongly Agree	Agree	Neutral	Disagree	Strongly Disagree	No Response
7. The NGO/CBO actively uses the pictorial micro-planning tool [AC2]	57%	43%	0%	0%	0%	0%

[AC3: Extensive onsite supportive supervision provided by managers and technical area specialists]

	Regularly	Sometimes	Never	No Response
B.7 During the past year has the NGO/CBO received supervisory visits from DAPCU or SACS or TSU?	93%	7%	0%	0%
B.8 Do you find supervisory visits to be a good opportunity for you to discuss solutions to any problems you may face?	86%	14%	0%	0%
	Yes	No		No Response
B.9 * Has supervision of your work changed due to the transition?	75%	21%		4%
	The better	The worse	Made no difference	No Response
a. If YES, was this a change for?	90%	5%	5%	0%
	SACS	NGO/CBO	Other	No Response
b. If YES, who brought about this change?	95%	0%	5%	0%

By State	Andra Pradesh	Karnataka	Tamil Nadu	Maharashtra
B.7 During the past year has the NGO/CBO received supervisory visits from DAPCU or SACS or TSU: Regularly	11/11	4/5	3/4	8/8
B.8 Do you find supervisory visits to be a good opportunity for you to discuss solutions to any problems you may face: Regularly	11/11	4/5	1/4	8/8
B.9 * Has supervision of your work changed due to the transition: Yes	10/11	3/5	4/4	4/8
a. If YES, was this a change for: The better	10/10	2/3	4/4	3/4
b. If YES, who brought about this change: SACS	9/10	3/3	4/4	4/4

Perceptions	Strongly Agree	Agree	Neutral	Disagree	Strongly Disagree	No Response
8. There is extensive supportive supervision of our work provided by managers and technical area specialists [AC3]	36%	61%	4%	0%	0%	0%

[AC4: Rigorous performance monitoring of peer outreach workers by staff supervisors and community committee]

	Regularly	Sometimes	Never	No Response
B.10 Is the performance of peer outreach workers monitored rigorously?	96%	4%	0%	0%
	Yes	No		No Response
B.11 * Has performance monitoring of peer outreach workers changed due to the transition?	50%	46%		4%
	The better	The worse	Made no difference	No Response
a. If YES, was this a change for?	93%	7%	0%	0%
	SACS	NGO/CBO	Other	No Response
b. If YES, who brought about this change?	64%	29%	7%	0%

By State	Andra Pradesh	Karnataka	Tamil Nadu	Maharashtra
B.10 Is the performance of peer outreach workers monitored rigorously: Regularly	11/11	5/5	4/4	7/8
B.11 * Has performance monitoring of peer outreach workers changed due to the transition: Yes	6/11	2/5	4/4	2/8
a. If YES, was this a change for: The better	5/6	2/2	4/4	2/2
b. If YES, who brought about this change: SACS	5/6	1/2	2/4	1/2

Perceptions	Strongly Agree	Agree	Neutral	Disagree	Strongly Disagree	No Response
9. Performance monitoring of PEs by staff supervisors is rigorous [AC4]	46%	50%	4%	0%	0%	0%
10. Performance monitoring of ORWs by staff supervisors is rigorous [AC4]	57%	43%	0%	0%	0%	0%

Relationship with SACS

[AC5: Flexible management style that facilitates response to local needs]

	Regularly	Sometimes	Never	No Response
C.1 Has SACS provided any flexibility on budget, based on realities on-the-ground?	0%	46%	54%	0%
C.2 Does SACS allow any exceptions to operating norms (other than budget) such as the PE/ORW ratio, based on realities on-the-ground?	14%	54%	32%	0%
	Yes	No		No Response

C.3 * Do you find that the SACS is as flexible in its management style as the Avahan SLP?	43%	54%		4%

By State	Andra Pradesh	Karnataka	Tamil Nadu	Maharashtra
C.1 Has SACS provided any flexibility on budget, based on realities on-the-ground: Regularly	0/11	0/5	0/4	0/8
C.2 Does SACS allow any exceptions to operating norms (other than budget) such as the PE/ORW ratio, based on realities on-the-ground: Regularly	1/11	1/5	0/4	2/8
C.3 * Do you find that the SACS is as flexible in its management style as the Avahan SLP: Yes	3/11	4/5	0/4	5/8

Perceptions	Strongly Agree	Agree	Neutral	Disagree	Strongly Disagree	No Response
11. SACS makes exceptions to NACO norms to meet local needs [AC5]	18%	46%	7%	25%	4%	0%

[AC6: "Common Minimum Program" with Clinic Operational Guidelines and Standards and Treatment Guidelines to build a shared vision and define operating standards]

	Regularly	Sometimes	Never	No Response
C.4 Do the staff follow the clinic operational guidelines and treatment guidelines set by NACO?	100%	0%	0%	0%
	Yes	No		No Response
C.5 * Has the clarity of guidelines on clinical services changed due to the transition?	43%	54%		4%
	The better	The worse	Made no difference	No Response
a. If YES, was this a change for?	92%	0%	8%	0%
	SACS	NGO/CBO	Other	No Response
b. If YES, who brought about this change?	100%	0%	0%	0%

By State	Andra Pradesh	Karnataka	Tamil Nadu	Maharashtra
C.4 Do the staff follow the clinic operational guidelines and treatment guidelines set by NACO: Regularly	11/11	5/5	4/4	8/8
C.5 * Has the clarity of guidelines on clinical services changed due to the transition: Yes	5/11	3/5	0/4	4/8
a. If YES, was this a change for: The better	4/5	3/3	0/0	4/4
b. If YES, who brought about this change: SACS	5/5	3/3	0/0	4/4

Perceptions	Strongly Agree	Agree	Neutral	Disagree	Strongly Disagree	No Response
12. There are clearly defined norms and standards that govern the clinical aspects of our work [AC6]	46%	43%	4%	4%	4%	0%
13. There are clearly defined norms and standards that govern the operational aspects of our work [AC6]	43%	50%	0%	7%	0%	0%
14. All of us working at this TI have a good understanding of operating procedures and a shared vision of program aims [AC6]	46%	50%	4%	0%	0%	0%
15. The NGO/CBO and SACS share a common vision for HIV prevention [AC6]	86%	14%	0%	0%	0%	0%

[AC7: On-time, adequate and uninterrupted flow of funds and commodities to the grassroots level]

	Regularly	Sometimes	Never	No Response
C.6 During the past year, have you ever had any problem with cash flows from the SACS that has affected your operations?	14%	46%	39%	0%
	Yes	No		No Response
C.7 * Has there been any change in the amount of funds provided to the TI due to the transition?	75%	18%		7%
C.8 * Has there been any change due to the transition, in funds arriving on time at the TI?	43%	50%		7%

By State	Andra Pradesh	Karnataka	Tamil Nadu	Maharashtra
C.6 During the past year, have you ever had any problem with cash flows from the SACS that has affected your operations: Regularly	0/11	1/5	2/4	1/8
C.7 * Has there been any change in the amount of funds provided to the TI due to the transition: Yes	9/11	4/5	4/4	4/8
C.8 * Has there been any change due to the transition, in funds arriving on time at the TI: Yes	8/11	0/5	4/4	0/8

Perceptions	Strongly Agree	Agree	Neutral	Disagree	Strongly Disagree	No Response
17. Funds from SACS to the NGO/CBO is on-time and adequate [AC7]	25%	64%	0%	7%	4%	0%

	Regularly	Sometimes	Never	No Response
C.9 During the past year has your TI always had sufficient stock of commodities, such as condoms or medicines?	68%	29%	4%	0%
	Yes	No		No Response
C.10 * Has there been any change in the quantity of commodities supplied to the TI due to the transition?	29%	64%		7%
	The better	The worse	Made no difference	No Response
a. If YES, was this a change for?	75%	13%	13%	0%
	SACS	NGO/CBO	Other	No Response
b. If YES, who brought about this change?	100%	0%	0%	0%
	Yes	No		No Response
C.11 * Has there been any change in the supply chain of commodities to the TI due to the transition?	61%	32%		7%
	The better	The worse	Made no difference	No Response
a. If YES, was this a change for?	71%	12%	18%	0%
	SACS	NGO/CBO	Other	No Response
b. If YES, who brought about this change?	100%	0%	0%	0%

By State	Andra Pradesh	Karnataka	Tamil Nadu	Maharashtra
C.9 During the past year has your TI always had sufficient stock of commodities, such as condoms or medicines: Regularly	5/11	4/5	3/4	7/8
C.10 * Has there been any change in the quantity of commodities supplied to the TI due to the transition: Yes	4/11	2/5	1/4	1/8
a. If YES, was this a change for: The better	4/4	1/2	1/1	0/1
b. If YES, who brought about this change: SACS	4/4	2/2	1/1	1/1
C.11 * Has there been any change in the supply chain of commodities to the TI due to the transition: Yes	9/11	2/5	4/4	2/8
a. If YES, was this a change for: The better	8/9	1/2	2/4	1/2
b. If YES, who brought about this change: SACS	9/9	2/2	4/4	2/2

Perceptions	Strongly Agree	Agree	Neutral	Disagree	Strongly Disagree	No Response
16. Commodities from SACS to the NGO/CBO is on-time and adequate [AC7]	21%	71%	0%	7%	0%	0%

[AC8: Support to service delivery through strong advocacy programs at national and state level]

	Regularly	Sometimes	Never	No Response
C.12 Do you find that SACS/NACO advocates on behalf of HRG programs?	39%	25%	36%	0%
	Yes	No		No Response
C.13 * Has the relationship between HRGs and local police changed due to the transition?	64%	32%		4%
C.14 * Has the relationship between HRGs and service providers changed due to the transition?	64%	32%		4%

By State	Andra Pradesh	Karnataka	Tamil Nadu	Maharashtra
C.12 Do you find that SACS/NACO advocates on behalf of HRG programs: Regularly	3/11	4/5	0/4	4/8
C.13 * Has the relationship between HRGs and local police changed due to the transition: Yes	9/11	2/5	4/4	3/8
C.14 * Has the relationship between HRGs and service providers changed due to the transition: Yes	10/11	2/5	4/4	2/8

Perceptions	Strongly Agree	Agree	Neutral	Disagree	Strongly Disagree	No Response
18. SACS is a good advocate for HRG programming [AC8]	21%	57%	11%	7%	4%	0%

Community activities

[AC9: Community-led crisis response management]

	Regularly	Sometimes	Never	No Response
D.1 Do you find that the crisis response system works?	86%	11%	4%	0%
	Yes	No		No Response
D.2 * Has crisis response management changed due to the transition?	36%	57%		7%
	The better	The worse	Made no difference	No Response
a. If YES, was this a change for?	80%	20%	0%	0%
	SACS	NGO/CBO	Other	No Response
b. If YES, who brought about this change?	100%	0%	0%	0%

By State	Andra Pradesh	Karnataka	Tamil Nadu	Maharashtra
D.1 Do you find that the crisis response system works: Regularly	10/11	4/5	3/4	7/8
D.2 * Has crisis response management changed due to the transition: Yes	2/11	3/5	2/4	3/8
a. If YES, was this a change for: The better	2/2	3/3	2/2	1/3
b. If YES, who brought about this change: SACS	2/2	3/3	2/2	3/3

Perceptions	Strongly Agree	Agree	Neutral	Disagree	Strongly Disagree	No Response
19. The crisis response system is led by the community [AC9]	46%	39%	11%	4%	0%	0%

[AC10: Strong focus on fostering community groups and organizations]

	Regularly	Sometimes	Never	No Response
D.3 Have you supported community groups and organizations?	82%	14%	4%	0%
	Yes	No		No Response
D.4 Have any of the community groups and organizations secured other sources of funding?	64%	36%		0%
D.5 * Has the focus on supporting community groups changed due to the transition?	36%	57%		7%
	The better	The worse	Made no difference	No Response
a. If YES, was this a change for?	60%	10%	30%	0%
	SACS	NGO/CBO	Other	No Response
b. If YES, who brought about this change?	80%	20%	0%	0%

By State	Andra Pradesh	Karnataka	Tamil Nadu	Maharashtra
D.3 Have you supported community groups and organizations: Regularly	7/11	4/5	4/4	8/8
D.4 Have any of the community groups and organizations secured other sources of funding: Yes	4/11	3/5	3/4	8/8
D.5 * Has the focus on supporting community groups changed due to the transition: Yes	5/11	0/5	2/4	3/8
a. If YES, was this a change for: The better	3/5	0/0	2/2	1/8
b. If YES, who brought about this change: SACS	4/5	0/0	1/2	3/3

Perceptions	Strongly Agree	Agree	Neutral	Disagree	Strongly Disagree	No Response
20. The NGO/CBO has a strong focus on fostering community groups and organizations [AC10]	61%	36%	4%	0%	0%	0%
21. Community groups have found alternative sources of financial support [AC10]	43%	36%	11%	11%	0%	0%

[AC11: Committees of community members that oversee the program]

	Regularly	Sometimes	Never	No Response
D.6 Does a committee of community members oversee the program?	86%	11%	4%	0%
	Yes	No		No Response
D.7 During the past year, has the committee of community members made any recommendations for program change?	54%	46%		0%
D.8 * Has there been a change, due to the transition, in oversight of the program by committees of community members?	36%	57%		7%
	The better	The worse	Made no difference	No Response
a. If YES, was this a change for?	90%	10%	0%	0%
	SACS	NGO/CBO	Other	No Response
b. If YES, who brought about this change?	80%	20%	0%	0%

By State	Andra Pradesh	Karnataka	Tamil Nadu	Maharashtra
D.6 Does a committee of community members oversee the program: Regularly	10/11	5/5	2/4	7/8
D.7 During the past year, has the committee of community members made any recommendations for program change: Yes	4/11	3/5	4/4	4/8
D.8 * Has there been a change, due to the transition, in oversight of the program by committees of community members: Yes	2/11	3/5	4/4	1/8
a. If YES, was this a change for: The better	2/2	3/3	4/4	0/1
b. If YES, who brought about this change: SACS	2/2	1/3	4/4	1/1

Perceptions	Strongly Agree	Agree	Neutral	Disagree	Strongly Disagree	No Response
22. Committees of community members oversee the program [AC11]	36%	57%	7%	0%	0%	0%

Training & Program coverage

[AC12: Need based systematic training to enhance peer outreach workers' skills and leadership]

	Regularly	Sometimes	Never	No Response
E.1 Are the training needs of PEs and ORWs assessed?	96%	4%	0%	0%
E.2 Do PEs and ORWs receive skills and leadership training (beyond general orientation training)?	46%	46%	7%	0%
	Yes	No		No Response
E.3 * Has training for PEs or ORWs changed due to the transition?	64%	32%		4%
	The better	The worse	Made no difference	No Response
a. If YES, was this a change for?	56%	17%	28%	0%
	SACS	NGO/CBO	Other	No Response
b. If YES, who brought about this change?	100%	0%	0%	0%

By State	Andra Pradesh	Karnataka	Tamil Nadu	Maharashtra
E.1 Are the training needs of PEs and ORWs assessed: Regularly	11/11	5/5	4/4	7/8
E.2 Do PEs and ORWs receive skills and leadership training (beyond general orientation training): Regularly	2/11	5/5	0/4	6/8
E.3 * Has training for PEs or ORWs changed due to the transition: Yes	8/11	3/5	3/4	4/8
a. If YES, was this a change for: The better	4/8	2/3	3/3	1/4
b. If YES, who brought about this change: SACS	8/8	3/3	3/3	4/4

Perceptions	Strongly Agree	Agree	Neutral	Disagree	Strongly Disagree	No Response
23. The NGO/CBO provides training to PEs and ORWs to enhance their skills and leadership [AC12]	46%	50%	4%	0%	0%	0%

[AC13: Saturation coverage of even smaller pockets of HRGs]

	Regularly	Sometimes	Never	No Response
E.4 Do you plan for saturated coverage of small pockets of HRGs?	89%	11%	0%	0%
	Yes	No		No Response
E.5 * Has coverage of smaller pockets of HRG changed due to the transition?	29%	64%		7%

	The better	The worse	Made no difference	No Response
a. If YES, was this a change for?	50%	50%	0%	0%
	SACS	NGO/CBO	Other	No Response
b. If YES, who brought about this change?	100%	0%	0%	0%

By State	Andra Pradesh	Karnataka	Tamil Nadu	Maharashtra
E.4 Do you plan for saturated coverage of small pockets of HRGs: Regularly	10/11	5/5	3/4	7/8
E.5 * Has coverage of smaller pockets of HRG changed due to the transition: Yes	1/11	1/5	2/4	4/8
a. If YES, was this a change for: The better	1/1	1/1	2/2	0/4
b. If YES, who brought about this change: SACS	1/1	1/1	2/2	4/4

Perceptions	Strongly Agree	Agree	Neutral	Disagree	Strongly Disagree	No Response
24. The NGO/CBO has high coverage even in smaller pockets of HRG [AC13]	50%	43%	4%	4%	0%	0%
