# Supplementary material for: Transitioning a Large Scale HIV/AIDS Prevention Program to Local Stakeholders: Findings from the Avahan Transition Evaluation
Source: PLoS One. 2015 Sep 1;10(9):e0136177. doi: 10.1371/journal.pone.0136177 (PMC4556643; doi:10.1371/journal.pone.0136177)
Supplement: S3 Table — (PDF) [file pone.0136177.s003.pdf]

WP 4: Institutionalization Study

<u>Quantitative Results from Round 2</u>

Study Overview

This study component focused on how well Avahan characteristics have become incorporated or 'institutionalized' by the Targeted Interventions (TIs) for high risk groups (HRGs) post-transition, from Avahan management and funding to that of State AIDS Control Societies (SACS). Round 2 of the survey was conducted between April - June 2013 among Project Directors (PD) or Coordinators (PC) of all TIs that transitioned in April 2014 (n=42), across 4 states.

	Inst. Round 2	TR Round 2*		
No. of TIs	42	53		
	Andra Pradesh	Karnataka	Tamil Nadu	Maharashtra
In which state are you located?	13	12	4	13
	FSW	MSM	Both	
For whom do you provide services?	18	8	16	
	NGO	CBO		
Are you a NGO or CBO?	30	12		
	Not Split	Split		
Did the TI Split due to transition?	34	8		

*We interviewed 53 TIs for Transition Readiness (TR) Study Round 2 and 42 TIs for this study. 10 TIs were not included in the Institutionalization study because they did not transition in April 2012, with concern that they may not have had time to institutionalize. One TI was lost to follow-up despite multiple efforts to interview.

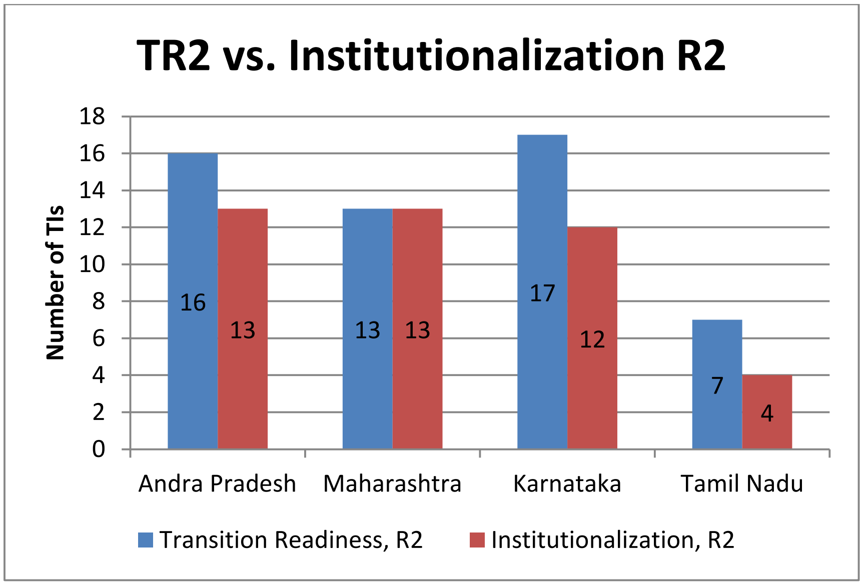

Transition Timing & Preparation

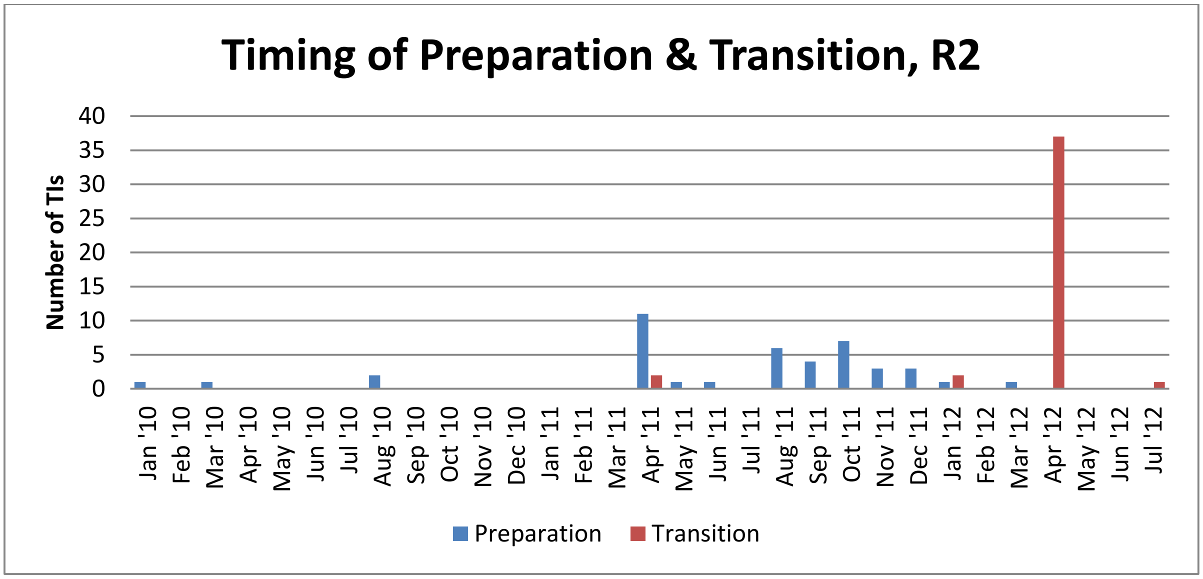

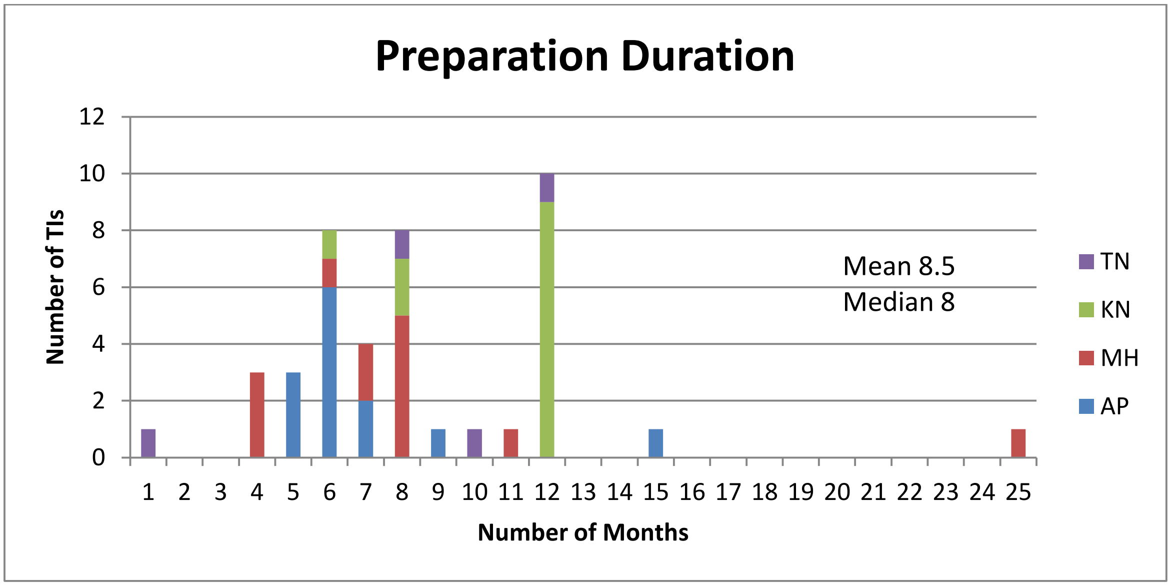

Transition Experience

Perceptions	Strongly Agree	Agree	Neutral	Disagree	Strongly Disagree	No Response
1. Overall, the transition experience went smoothly	43%	50%	2%	2%	2%	0%
2. The overall program has changed significantly as compared to pre-transition	31%	38%	17%	14%	0%	0%

3. SACS has the same or a higher level of commitment toward the program as compared to Avahan	29%	48%	14%	7%	0%	2%
4. The NGO/CBO has received support from the SLP after the transition	45%	43%	7%	5%	0%	0%

Data use & Supervision

[AC1: Active use of data at all levels for planning and regular review of program delivery]

	Regularly	Sometimes	Never	No Response
B.1 Does your NGO/CBO actively use data for program planning	100%	0%	0%	0%
	Yes	No		No Response
B.2 * Has the use of data for planning changed due to the transition?	62%	38%		0%
	The better	The worse	Made no difference	No Response
a. If YES, was this a change for?	88%	8%	4%	0%
	SACS	NGO/CBO	Other	No Response
b. If YES, who brought about this change?	92%	4%	4%	0%

By State (# of TIs)	Andra Pradesh	Karnataka	Tamil Nadu	Maharashtra
B.1 Does your NGO/CBO actively use data for program planning: Regularly	13/13	11/12	4/4	13/13
B.2 * Has the use of data for planning changed due to the transition: Yes	7/13	12/12	2/4	5/13
a. If YES, was this a change for: The better	6/7	10/12	2/2	5/5
b. If YES, who brought about this change: SACS	6/7	11/12	2/2	5/5

Perceptions	Strongly Agree	Agree	Neutral	Disagree	Strongly Disagree	No Response
5. The NGO/CBO actively uses data for program planning [AC1]	67%	31%	2%	0%	0%	0%

	Regularly	Sometimes	Never	No Response
B.3 Does your NGO/CBO actively use data to monitor progress in the program?	98%	2%	0%	0%
	Yes	No		No Response
B.4 * Has the use of data to monitor progress changed due to the transition?	60%	40%		0%
	The better	The worse	Made no	No Response

			difference	
a. If YES, was this a change for?	88%	8%	4%	0%
	SACS	NGO/CBO	Other	No Response
b. If YES, who brought about this change?	80%	16%	4%	0%

By State	Andra Pradesh	Karnataka	Tamil Nadu	Maharashtra
B.3 Does your NGO/CBO actively use data to monitor progress in the program: Regularly	13/13	11/12	4/4	13/13
B.4 * Has the use of data to monitor progress changed due to the transition: Yes	6/13	8/12	1/4	10/13
a. If YES, was this a change for: The better	4/6	7/8	1/1	10/10
b. If YES, who brought about this change: SACS	3/6	7/8	1/1	9/10

Perceptions	Strongly Agree	Agree	Neutral	Disagree	Strongly Disagree	No Response
6. The NGO/CBO actively uses data to monitor program progress [AC1]	62%	36%	0%	0%	2%	0%

[AC2: Pictorial micro-planning tool for peer outreach workers]

	Regularly	Sometimes	Never	No Response
B.5 Do peer outreach workers use pictorial micro-planning to facilitate their mapping of most at risk populations?	95%	5%	0%	0%
	Yes	No		No Response
B.6 * Has the use of pictorial micro-planning tool changed due to the transition?	40%	60%		0%
	The better	The worse	Made no difference	No Response
a. If YES, was this a change for?	82%	12%	6%	0%
	SACS	NGO/CBO	Other	No Response
b. If YES, who brought about this change?	88%	12%	0%	0%

By State	Andra Pradesh	Karnataka	Tamil Nadu	Maharashtra
B.5 Do peer outreach workers use pictorial micro-planning to facilitate their mapping of most at risk populations: Regularly	13/13	11/12	4/4	12/13
B.6 * Has the use of pictorial micro-planning tool changed due to the transition: Yes	3/13	7/12	2/4	5/13
a. If YES, was this a change for: The better	3/3	5/7	2/2	4/5
b. If YES, who brought about this change: SACS	3/3	7/7	2/2	3/5

Perceptions	Strongly Agree	Agree	Neutral	Disagree	Strongly Disagree	No Response
7. The NGO/CBO actively uses the pictorial micro-planning tool [AC2]	69%	26%	2%	2%	0%	0%

[AC3: Extensive onsite supportive supervision provided by managers and technical area specialists]

	Regularly	Sometimes	Never	No Response
B.7 During the past year has the NGO/CBO received supervisory visits from DAPCU or SACS or TSU?	88%	12%	0%	0%
B.8 Do you find supervisory visits to be a good opportunity for you to discuss solutions to any problems you may face?	90%	7%	2%	0%
	Yes	No		No Response
B.9 * Has supervision of your work changed due to the transition?	60%	40%		0%
	The better	The worse	Made no difference	No Response
a. If YES, was this a change for?	76%	12%	12%	0%
	SACS	NGO/CBO	Other	No Response
b. If YES, who brought about this change?	96%	0%	4%	0%

By State	Andra Pradesh	Karnataka	Tamil Nadu	Maharashtra
B.7 During the past year has the NGO/CBO received supervisory visits from DAPCU or SACS or TSU: Regularly	13/13	7/12	4/4	13/13
B.8 Do you find supervisory visits to be a good opportunity for you to discuss solutions to any problems you may face: Regularly	12/13	11/12	4/4	11/13
B.9 * Has supervision of your work changed due to the transition: Yes	8/13	10/12	2/4	5/13
a. If YES, was this a change for: The better	4/8	8/10	2/2	5/5
b. If YES, who brought about this change: SACS	7/8	10/10	2/2	5/5

Perceptions	Strongly Agree	Agree	Neutral	Disagree	Strongly Disagree	No Response
8. There is extensive supportive supervision of our work provided by managers and technical area specialists [AC3]	67%	21%	5%	7%	0%	0%

[AC4: Rigorous performance monitoring of peer outreach workers by staff supervisors and community committee]

	Regularly	Sometimes	Never	No Response
B.10 Is the performance of peer outreach workers monitored rigorously?	100%	0%	0%	0%
	Yes	No		No Response
B.11 * Has performance monitoring of peer outreach workers changed due to the transition?	64%	36%		0%
	The better	The worse	Made no difference	No Response
a. If YES, was this a change for?	93%	4%	4%	0%
	SACS	NGO/CBO	Other	No Response
b. If YES, who brought about this change?	78%	19%	4%	0%

By State	Andra Pradesh	Karnataka	Tamil Nadu	Maharashtra
B.10 Is the performance of peer outreach workers monitored rigorously: Regularly	13/13	12/12	4/4	13/13
B.11 * Has performance monitoring of peer outreach workers changed due to the transition: Yes	8/13	8/12	2/4	9/13
a. If YES, was this a change for: The better	7/8	7/8	2/2	9/9
b. If YES, who brought about this change: SACS	7/8	7/8	1/2	6/9

Perceptions	Strongly Agree	Agree	Neutral	Disagree	Strongly Disagree	No Response
9. Performance monitoring of PEs by staff supervisors is rigorous [AC4]	62%	33%	2%	2%	0%	0%
10. Performance monitoring of ORWs by staff supervisors is rigorous [AC4]	69%	29%	0%	2%	0%	0%

Relationship with SACS

[AC5: Flexible management style that facilitates response to local needs]

	Regularly	Sometimes	Never	No Response
C.1 Has SACS provided any flexibility on budget, based on realities on-the-ground?	10%	19%	71%	0%
C.2 Does SACS allow any exceptions to operating norms (other than budget) such as the PE/ORW ratio, based on realities on-the-ground?	26%	40%	33%	0%
	Yes	No		No Response

C.3 * Do you find that the SACS is as flexible in its management style as the Avahan SLP?	40%	60%		0%

By State	Andra Pradesh	Karnataka	Tamil Nadu	Maharashtra
C.1 Has SACS provided any flexibility on budget, based on realities on-the-ground: Regularly	0/13	2/12	1/4	1/13
C.2 Does SACS allow any exceptions to operating norms (other than budget) such as the PE/ORW ratio, based on realities on-the-ground: Regularly	6/13	1/12	1/4	3/13
C.3 * Do you find that the SACS is as flexible in its management style as the Avahan SLP: Yes	5/13	4/12	3/4	5/13

Perceptions	Strongly Agree	Agree	Neutral	Disagree	Strongly Disagree	No Response
11. SACS makes exceptions to NACO norms to meet local needs [AC5]	12%	29%	17%	24%	19%	0%

[AC6: "Common Minimum Program" with Clinic Operational Guidelines and Standards and Treatment Guidelines to build a shared vision and define operating standards]

	Regularly	Sometimes	Never	No Response
C.4 Do the staff follow the clinic operational guidelines and treatment guidelines set by NACO?	100%	0%	0%	0%
	Yes	No		No Response
C.5 * Has the clarity of guidelines on clinical services changed due to the transition?	26%	74%		0%
	The better	The worse	Made no difference	No Response
a. If YES, was this a change for?	91%	9%	0%	0%
	SACS	NGO/CBO	Other	No Response
b. If YES, who brought about this change?	100%	0%	0%	0%

By State	Andra Pradesh	Karnataka	Tamil Nadu	Maharashtra
C.4 Do the staff follow the clinic operational guidelines and treatment guidelines set by NACO: Regularly	13/13	12/12	4/4	13/13
C.5 * Has the clarity of guidelines on clinical services changed due to the transition: Yes	2/13	8/12	1/4	0/13
a. If YES, was this a change for: The better	2/2	7/8	1/1	0/0
b. If YES, who brought about this change: SACS	2/2	8/8	1/1	0/0

Perceptions	Strongly Agree	Agree	Neutral	Disagree	Strongly Disagree	No Response
12. There are clearly defined norms and standards that govern the clinical aspects of our work [AC6]	52%	40%	7%	0%	0%	0%
13. There are clearly defined norms and standards that govern the operational aspects of our work [AC6]	67%	26%	5%	2%	0%	0%
14. All of us working at this TI have a good understanding of operating procedures and a shared vision of program aims [AC6]	71%	26%	2%	0%	0%	0%
15. The NGO/CBO and SACS share a common vision for HIV prevention [AC6]	83%	14%	0%	0%	0%	2%

[AC7: On-time, adequate and uninterrupted flow of funds and commodities to the grassroots level]

	Regularly	Sometimes	Never	No Response
C.6 During the past year, have you ever had any problem with cash flows from the SACS that has affected your operations?	7%	43%	50%	0%
	Yes	No		No Response
C.7 * Has there been any change in the amount of funds provided to the TI due to the transition?	60%	40%		0%
C.8 * Has there been any change due to the transition, in funds arriving on time at the TI?	62%	38%		0%

By State	Andra Pradesh	Karnataka	Tamil Nadu	Maharashtra
C.6 During the past year, have you ever had any problem with cash flows from the SACS that has affected your operations: Regularly	2/13	1/12	0/4	0/13
C.7 * Has there been any change in the amount of funds provided to the TI due to the transition: Yes	12/13	9/12	2/4	2/13
C.8 * Has there been any change due to the transition, in funds arriving on time at the TI: Yes	9/13	10/12	3/4	4/13

Perceptions	Strongly Agree	Agree	Neutral	Disagree	Strongly Disagree	No Response
17. Funds from SACS to the NGO/CBO is on-time and adequate [AC7]	26%	26%	29%	14%	2%	2%

	Regularly	Sometimes	Never	No Response
C.9 During the past year has your TI always had sufficient stock of commodities, such as condoms or medicines?	69%	31%	0%	0%
	Yes	No		No Response
C.10 * Has there been any change in the quantity of commodities supplied to the TI due to the transition?	36%	64%		0%
	The better	The worse	Made no difference	No Response
a. If YES, was this a change for?	40%	40%	20%	0%
	SACS	NGO/CBO	Other	No Response
b. If YES, who brought about this change?	100%	0%	0%	0%
	Yes	No		No Response
C.11 * Has there been any change in the supply chain of commodities to the TI due to the transition?	64%	36%		0%
	The better	The worse	Made no difference	No Response
a. If YES, was this a change for?	41%	15%	44%	0%
	SACS	NGO/CBO	Other	No Response
b. If YES, who brought about this change?	96%	4%	0%	0%

By State	Andra Pradesh	Karnataka	Tamil Nadu	Maharashtra
C.9 During the past year has your TI always had sufficient stock of commodities, such as condoms or medicines: Regularly	5/13	9/12	4/4	11/13
C.10 * Has there been any change in the quantity of commodities supplied to the TI due to the transition: Yes	4/13	8/12	0/4	3/13
a. If YES, was this a change for: The better	2/4	3/8	0/0	1/3
b. If YES, who brought about this change: SACS	4/4	8/8	0/0	3/3
C.11 * Has there been any change in the supply chain of commodities to the TI due to the transition: Yes	12/13	8/12	2/4	5/13
a. If YES, was this a change for: The better	6/12	3/8	0/2	2/5
b. If YES, who brought about this change: SACS	12/12	8/8	2/2	4/5

Perceptions	Strongly Agree	Agree	Neutral	Disagree	Strongly Disagree	No Response
16. Commodities from SACS to the NGO/CBO is on-time and adequate [AC7]	33%	31%	17%	17%	0%	2%

[AC8: Support to service delivery through strong advocacy programs at national and state level]

	Regularly	Sometimes	Never	No Response
C.12 Do you find that SACS/NACO advocates on behalf of HRG programs?	38%	24%	38%	0%
	Yes	No		No Response
C.13 * Has the relationship between HRGs and local police changed due to the transition?	33%	67%		0%
C.14 * Has the relationship between HRGs and service providers changed due to the transition?	55%	45%		0%

By State	Andra Pradesh	Karnataka	Tamil Nadu	Maharashtra
C.12 Do you find that SACS/NACO advocates on behalf of HRG programs: Regularly	0/13	10/12	2/4	4/13
C.13 * Has the relationship between HRGs and local police changed due to the transition: Yes	3/13	3/12	3/4	5/13
C.14 * Has the relationship between HRGs and service providers changed due to the transition: Yes	8/13	9/12	3/4	3/13

Perceptions	Strongly Agree	Agree	Neutral	Disagree	Strongly Disagree	No Response
18. SACS is a good advocate for HRG programming [AC8]	48%	36%	10%	2%	2%	2%

Community activities

[AC9: Community-led crisis response management]

	Regularly	Sometimes	Never	No Response
D.1 Do you find that the crisis response system works?	86%	14%	0%	0%
	Yes	No		No Response
D.2 * Has crisis response management changed due to the transition?	31%	69%		0%
	The better	The worse	Made no difference	No Response
a. If YES, was this a change for?	54%	31%	15%	0%
	SACS	NGO/CBO	Other	No Response
b. If YES, who brought about this change?	77%	15%	8%	0%

By State	Andra Pradesh	Karnataka	Tamil Nadu	Maharashtra
D.1 Do you find that the crisis response system works: Regularly	12/13	11/12	4/4	9/13
D.2 * Has crisis response management changed due to the transition: Yes	5/13	3/12	2/4	3/13
a. If YES, was this a change for: The better	1/5	2/3	1/2	3/3
b. If YES, who brought about this change: SACS	5/5	2/3	1/2	2/3

Perceptions	Strongly Agree	Agree	Neutral	Disagree	Strongly Disagree	No Response
19. The crisis response system is led by the community [AC9]	48%	45%	7%	0%	0%	0%

[AC10: Strong focus on fostering community groups and organizations]

	Regularly	Sometimes	Never	No Response
D.3 Have you supported community groups and organizations?	74%	26%	0%	0%
	Yes	No		No Response
D.4 Have any of the community groups and organizations secured other sources of funding?	45%	52%		2%
D.5 * Has the focus on supporting community groups changed due to the transition?	26%	74%		0%
	The better	The worse	Made no difference	No Response
a. If YES, was this a change for?	73%	9%	18%	0%
	SACS	NGO/CBO	Other	No Response
b. If YES, who brought about this change?	73%	9%	18%	0%

By State	Andra Pradesh	Karnataka	Tamil Nadu	Maharashtra
D.3 Have you supported community groups and organizations: Regularly	10/13	9/12	3/4	9/13
D.4 Have any of the community groups and organizations secured other sources of funding: Yes	5/13	4/12	3/4	7/13
D.5 * Has the focus on supporting community groups changed due to the transition: Yes	3/13	5/12	1/4	2/13
a. If YES, was this a change for: The better	3/3	2/5	1/1	2/2
b. If YES, who brought about this change: SACS	2/3	5/5	0/1	1/2

Perceptions	Strongly Agree	Agree	Neutral	Disagree	Strongly Disagree	No Response
20. The NGO/CBO has a strong focus on fostering community groups and organizations [AC10]	57%	40%	2%	0%	0%	0%
21. Community groups have found alternative sources of financial support [AC10]	19%	29%	21%	17%	12%	2%

[AC11: Committees of community members that oversee the program]

	Regularly	Sometimes	Never	No Response
D.6 Does a committee of community members oversee the program?	69%	21%	7%	2%
	Yes	No		No Response
D.7 During the past year, has the committee of community members made any recommendations for program change?	60%	38%		2%
D.8 * Has there been a change, due to the transition, in oversight of the program by committees of community members?	19%	81%		0%
	The better	The worse	Made no difference	No Response
a. If YES, was this a change for?	88%	13%	0%	0%
	SACS	NGO/CBO	Other	No Response
b. If YES, who brought about this change?	88%	13%	0%	0%

By State	Andra Pradesh	Karnataka	Tamil Nadu	Maharashtra
D.6 Does a committee of community members oversee the program: Regularly	12/13	5/12	3/4	9/13
D.7 During the past year, has the committee of community members made any recommendations for program change: Yes	6/13	9/12	2/4	8/13
D.8 * Has there been a change, due to the transition, in oversight of the program by committees of community members: Yes	0/13	6/12	2/4	0/13
a. If YES, was this a change for: The better	0/0	6/6	1/2	0/0
b. If YES, who brought about this change: SACS	0/0	5/6	2/2	0/0

Perceptions	Strongly Agree	Agree	Neutral	Disagree	Strongly Disagree	No Response
22. Committees of community members oversee the program [AC11]	26%	50%	17%	7%	0%	0%

Training & Program coverage

[AC12: Need based systematic training to enhance peer outreach workers' skills and leadership]

	Regularly	Sometimes	Never	No Response
E.1 Are the training needs of PEs and ORWs assessed?	90%	5%	5%	0%
E.2 Do PEs and ORWs receive skills and leadership training (beyond general orientation training)?	57%	33%	10%	0%
	Yes	No		No Response
E.3 * Has training for PEs or ORWs changed due to the transition?	55%	45%		0%
	The better	The worse	Made no difference	No Response
a. If YES, was this a change for?	48%	26%	26%	0%
	SACS	NGO/CBO	Other	No Response
b. If YES, who brought about this change?	96%	4%	0%	0%

By State	Andra Pradesh	Karnataka	Tamil Nadu	Maharashtra
E.1 Are the training needs of PEs and ORWs assessed: Regularly	13/13	11/12	4/4	10/13
E.2 Do PEs and ORWs receive skills and leadership training (beyond general orientation training): Regularly	4/13	9/12	3/4	8/13
E.3 * Has training for PEs or ORWs changed due to the transition: Yes	11/13	8/12	3/4	1/13
a. If YES, was this a change for: The better	4/11	4/8	2/3	1/1
b. If YES, who brought about this change: SACS	11/11	8/8	2/3	1/1

Perceptions	Strongly Agree	Agree	Neutral	Disagree	Strongly Disagree	No Response
23. The NGO/CBO provides training to PEs and ORWs to enhance their skills and leadership [AC12]	60%	38%	2%	0%	0%	0%

[AC13: Saturation coverage of even smaller pockets of HRGs]

	Regularly	Sometimes	Never	No Response
E.4 Do you plan for saturated coverage of small pockets of HRGs?	95%	5%	0%	0%
	Yes	No		No Response
E.5 * Has coverage of smaller pockets of HRG changed due to the transition?	14%	86%		0%

	The better	The worse	Made no difference	No Response
a. If YES, was this a change for?	67%	33%	0%	0%
	SACS	NGO/CBO	Other	No Response
b. If YES, who brought about this change?	83%	17%	0%	0%

By State	Andra Pradesh	Karnataka	Tamil Nadu	Maharashtra
E.4 Do you plan for saturated coverage of small pockets of HRGs: Regularly	12/13	12/12	4/4	12/13
E.5 * Has coverage of smaller pockets of HRG changed due to the transition: Yes	0/13	3/12	2/4	1/13
a. If YES, was this a change for: The better	0/0	1/3	2/2	1/1
b. If YES, who brought about this change: SACS	0/0	3/3	2/2	0/1

Perceptions	Strongly Agree	Agree	Neutral	Disagree	Strongly Disagree	No Response
24. The NGO/CBO has high coverage even in smaller pockets of HRG [AC13]	57%	36%	7%	0%	0%	0%
